# Computational selection and prioritization of candidate genes for Fetal Alcohol Syndrome

**DOI:** 10.1186/1471-2164-8-389

**Published:** 2007-10-25

**Authors:** Zané Lombard, Nicki Tiffin, Oliver Hofmann, Vladimir B Bajic, Winston Hide, Michèle Ramsay

**Affiliations:** 1 Division of Human Genetics, National Health Laboratory Service & School of Pathology, University of the Witwatersrand, Johannesburg, 2001, South Africa; 2 South African National Bioinformatics Institute (SANBI) Research Group, University of the Western Cape, Bellville, 7530, South Africa; 3 Division of Human Genetics, University of Cape Town, Cape Town, 8001, South Africa

## Abstract

**Background:**

Fetal alcohol syndrome (FAS)  is a serious global health problem and is observed at high frequencies in certain South African communities. Although *in utero *alcohol exposure is the primary trigger, there is evidence for genetic- and other susceptibility factors in FAS development. No genome-wide association or linkage studies have been performed for FAS, making computational selection and -prioritization of candidate disease genes an attractive approach.

**Results:**

10174 Candidate genes were initially selected from the whole genome using a previously described method, which selects candidate genes according to their expression in disease-affected tissues. Hereafter candidates were prioritized for experimental investigation by investigating criteria pertinent to FAS and binary filtering. 29 Criteria were assessed by mining various database sources to populate criteria-specific gene lists. Candidate genes were then prioritized for experimental investigation using a binary system that assessed the criteria gene lists against the candidate list, and candidate genes were scored accordingly. A group of 87 genes was prioritized as candidates and for future experimental validation. The validity of the binary prioritization method was assessed by investigating the protein-protein interactions, functional enrichment and common promoter element binding sites of the top-ranked genes.

**Conclusion:**

This analysis highlighted a list of strong candidate genes from the TGF-β, MAPK and Hedgehog signalling pathways, which are all integral to fetal development and potential targets for alcohol's teratogenic effect. We conclude that this novel bioinformatics approach effectively prioritizes credible candidate genes for further experimental analysis.

## Background

### Case Study Disease: Fetal Alcohol Syndrome

Fetal alcohol syndrome (FAS) is the most common preventable cause of mental retardation globally, and is a serious public health problem in South Africa [1]. The range of prevalence rates reported in two different primary school cohorts from this community were 65.2–74.2 per 1 000 [2] and 68.0–89.2 per 1000 [1] respectively. This rate is alarmingly higher than the average observed for the developed world of 0.97 per 1000 live births [3].

The teratogenic effect of alcohol is well established and exposure to alcohol *in utero *is known to result in a widely variable phenotype. Fetal alcohol spectrum disorder (FASD) is an umbrella term used to describe the irreversible array of anomalies associated with *in utero *alcohol exposure [4]. These anomalies include prenatal and postnatal growth retardation, central nervous system (CNS) dysfunction, characteristic craniofacial malformation and other organ abnormalities [5-7]. The term FAS is a clinical description for children at the most severe end of the FASD spectrum, who display the full phenotype associated with *in utero *alcohol exposure.

Although alcohol consumption during pregnancy is the primary trigger for the presentation of FAS, the exact mechanisms for alcohol-induced teratogenic effects have not been elucidated. Research has shown that secondary factors, like genetic, epigenetic and environmental factors influence the outcome and severity of the disorder. Furthermore, a dose- and time-dependant relationship has been observed, where exposure to higher concentrations of alcohol at critical developmental stages resulted in more severe anomalies [8]. An association between a variable genetic background and FAS development is primarily supported by the observation that FAS does not occur in all children exposed to alcohol during the prenatal period [9]. This observation suggests that certain individuals may have a genetic predisposition to infliction of more severe damage by gestational alcohol consumption; and the varied phenotype observed in FASD may be a reflection of the varied susceptibility quotients in the genetic background of the individual. Streissguth and Dehaene [10] studied twin pairs with alcoholic mothers, and found the rate of concordance for FASD to be 100% for monozygotic twins, whereas digygotic twins showed only 64% concordance. Further support for the role of genetics in FAS development is obtained from animal model studies [11]. Several studies in different mouse strains have shown variation in the extent and pattern of alcohol-induced malformation, as well as behavioural outcome [12-15]. FAS can therefore be considered to be a multi-factorial or complex disease, suggesting that there are multiple genetic factors underlying susceptibility to FAS and the interactions between these factors as well as other factors are likely to be intricate.

### Disease gene identification for FAS

To date, no FAS family linkage studies or genome wide association studies have been performed. Linkage studies require large family samples and this poses a significant challenge. Countries with the highest FAS rates are mostly resource-poor, possibly contributing to the reason why such studies have not yet been performed. Furthermore, linkage studies have not proven to be particularly successful in discovering the genetic causes of complex diseases, the critical factor being the generally weak genotype-phenotype association in multi-factorial disorders [16].

Few candidate gene association studies investigating the effect of specific genetic polymorphisms on the risk of FAS development have been published. These studies have generally focused on the alcohol dehydrogenase enzyme family members and conflicting results have been obtained. Stoler *et al*. [17] observed that the absence of the ADH1B*3 allele was protective for fetal outcome, in conflict with two other studies showing the presence of this allele to be protective [18,19]. The ADH1B*2 allele has been proposed to play a possible protective role, or to be a marker for protection in the South African mixed-ancestry population [20]. However, the sample size for this association study was small, and results have not yet been replicated in other populations. Many other genes are likely to contribute towards the development of FAS and further investigation is required.

Candidate gene association studies remain the most practical and frequently employed approach in disease gene investigation for complex disorders. However, the main challenge when using this approach is to select suitable genes to test, especially for diseases with poorly understood aetiology. Recently, many computational candidate gene selection and -prioritization methods have been developed [21-31]. These tools aim to identify and prioritize putative disease genes by modelling specific characteristics of known disease genes, or by focusing on known disease features (such as gene expression profiles or phenotype). However, there is a vast quantity of information and data sources available currently, and it is expected that a tool with the flexibility to include a large array of data sources would positively aid disease gene discovery. The freely accessible tool Endeavour offers such an application [22]. This tool is based on the premise of ranking unknown candidate genes according to their similarity with a known set of training genes. In the absence of a linked genetic region (which is the case with FAS), all genes in the genome must be included as a starting point for candidate gene selection, which is not feasible when using this approach.

Convergent Functional Genomics (CFG) is an approach used to identify and prioritize candidate genes, which  relies on the cross-matching of animal model gene expression data with human genetic linkage data, as well as human tissue data and biological roles data [32,33] This approach has many parallels to the approach described in this paper, as it prescribes a Bayesian-like methodology of reducing uncertainty through the combination of multiple independent lines of evidence, each by itself lacking sufficient power to confirm that a gene is a putative candidate gene, to produce a short list of high probability candidate genes [32]. The approach of CFG relies principally on two lines of evidence – animal model data and human genetic linkage data. The approach we describe in this paper has the added advantage of allowing the inclusion of additional lines of evidence in the presence of limited expression studies in an animal model and the absence of FAS linkage studies.

Tiffin *et al*. [34] recently surveyed some of the methods for computational disease gene identification and concluded that using the methods in concert was more successful in prioritizing candidate genes for disease, than when each was used alone. This review additionally showed that using existing computational methods in concert highlighted potential candidates that are selected by a subset of methods and are missed by the other methods, depending on the type of data examined. This observation gives further evidence that the inclusion of more data sources may positively aid disease gene discovery.

In light of the current burden of FAS in many resource-poor communities and the inconclusive search for susceptibility genes, computational identification offers a novel and efficient approach to the identification of disease-causing genes. Initially we used the candidate gene selection method described by Tiffin *et al*. [31], but in the absence of a candidate genetic region, this method resulted in a large candidate gene list, as it relies on the selection of candidate disease genes only according to their expression profiles. This prompted us to devise a new prioritization method to rank genes from the candidate gene list for empirical investigation. The prioritization method described here is based on a simulation of a researcher's approach to selecting candidate disease genes. In this process, a variety of relevant database sources are mined for candidate genes that exhibit characteristics relevant to disease phenotype. Genes were prioritized based on binary evaluation, where genes were assessed using criteria pertinent to FAS to mine various database sources and to create criteria-specific gene lists. The validity of the binary prioritization method was assessed by investigating the protein-protein interactions, functional enrichment and common promoter element binding sites of the top-ranked genes.

## Results

### Integrated literature- and data mining for candidate gene selection

According to the method described by Tiffin *et al*. [31], Dragon Disease Explorer (DDE) was used to extract eVOC anatomical terms from the body of literature, where after they were used to extract candidate genes from the Ensembl database. This method extracted a list of 10174 genes, a reduction of 70.3% from the original 34294 genes in the Ensembl database.

### Binary filtering and prioritization of candidate genes

In order to select the most likely candidates from the initial candidate gene list, these genes were ranked according to the number of additional criteria (Table 1) they matched. The top-ranked genes (in ranked order) are shown in Table 2. *FGFR1 *was the top-ranked gene, present in 17 of the 29 criteria gene lists, followed by *MSX1*, present in 16 of the 29 criteria lists. *FGFR2, FOXG1B *and *HOXA1 *were present in 15 of the 29 criteria lists, followed by a group of 4, 17, 14 and 47 genes present in 14, 13, 12 and 11 criteria lists, respectively. This group of 87 genes was used as the prioritized candidate gene list for further analyses (see Additional file 1). This cut off (present in 11 of the 29 criteria lists) was used to select an appropriately-sized group of top-ranked genes.

**Table 1:** Summary of criteria used to create a binary grid

**CATEGORIES**				
**Cell type**	**Biological Process**	**Animal model homology**	**Phenotype simile**	**Imprinted genes**

Glial cell	Apoptosis	*Phenotype*	Mental Retardation	All known human
Neuron	Development	Growth	Microcephaly	imprinted genes
Fibroblast	Brain Development	Behaviour/Neurological	Craniofacial	
Neuroepithelium	Transport	Craniofacial	Hyperactivity	
	Signal Transduction	Nervous	Growth Retardation	
		Embryogenesis		
				
		*Timing*		
		Pre-Embryonic		
		Embryonic		
		Fetal		
				
		*Anatomy*		
		TS^1^8–9 Ectoderm		
		TS10–13 Neural Ectoderm		
		TS14–26 CNS		
		TS28 CNS		
		TS12–26 Head		
		TS20–26 Cranium		

^1^TS – Theiller stage: A term used to denote the stage of development of a mouse as described by Theiler in "The House Mouse: Atlas of Mouse Development" (Springer-Verlag, New York, 1989)

**Table 2:** Selected top-ranked candidate genes for FAS identified using binary matrix filtering

**Rank**	**Criteria matched**	**HGNC ID**	**Description**	**Location**	**Function**
1	17/29	*FGFR1*^1^	Fibroblast growth factor receptor 1	8p11.2	Involved in limb induction, play a role in bone elongation modulation
2	16/29	*MSX1*^2^	Msh homeobox homolog 1 gene	4p16.3-p16.1	Potential repressor function in cell cycle progression, transcription repressor
3	15/29	*FGFR2*^1^	Fibroblast growth factor receptor 2	10q26	Involved in vertebral development, important regulator of bone formation and osteoblast activity
4	15/29	*FOXG1B*	Forkhead box G1B	14q13	Embryonic transcriptional regulator, playing a critical role in brain development
5	15/29	*HOXA1*	Homeobox A1	7p15.3	Involved in the placement of hindbrain segments in the proper location along the anterior-posterior axis during development
6	14/29	*BMP4*^2,3^	Bone morphogenetic protein 4	14q22-q23	Regulating myogenesis through dosage-dependent PAX3 expression in pre-myogenic cells, inducing apoptosis and chondrogenesis in the chick limb bud
7	14/29	*FGFR3*^1^	Fibroblast growth factor receptor 3	4p16.3	Negative regulator of bone growth promotion, inhibition of chondrocyte proliferation and differentiation depending on devlopmental time
8	14/29	*GNAS*^2^	Gnas complex locus	20q13.2-q13.3	Involved as modulators or transducers in various transmembrane signaling systems primarily mediating the differential effects of parathyroid hormone
9	14/29	*PAX6*	Paired box gene 6	11p13	Key regulator of eye, pancreas, central nervous system development and regulator of glial precursors in the ventral neural tube

^1 ^Members of/linked to the MAPK signalling pathway

^2 ^Members of/linked to the TGF-β signalling pathway

^3^Members of/linked to the Hedgehog signalling pathway

Genes from the candidate gene lists that matched one or none of the criteria were considered to be unlikely candidates. Based on this premise, these 5055 genes (50%) from the candidate gene list were ranked as weak candidates. 87 Genes of the subset matching to no criteria were randomly selected for further analysis as a negative control set to assess the validity of the ranking method.

### Evaluation of biological significance of prioritized genes

#### Protein-protein interactions

**Figure 1:**
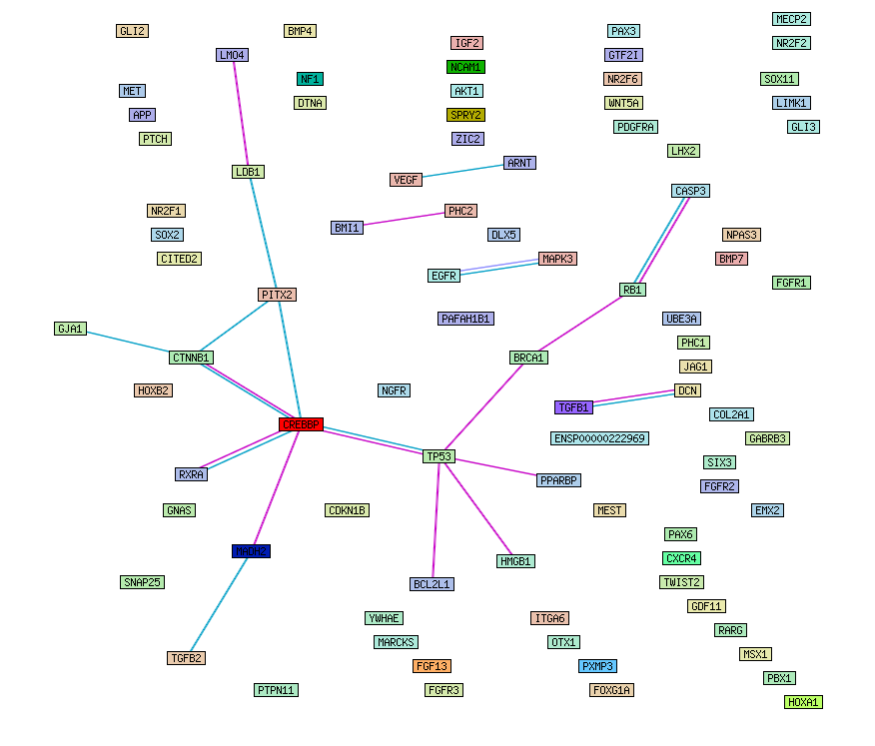
**The STRING network of known protein-protein interaction among the 78 top-ranked candidate genes for FAS**. The network edges represent the predicted functional associations. Each different colour represents a different line of evidence: pink = experimental data, light blue = homology analysis and turquoise = functional association.

The list of most likely candidate genes (top-ranked 87 genes), and unlikely candidates (randomly selected low-ranking 87 genes) were submitted to the STRING database (Search tool for the retrieval of interacting genes/proteins) [35] to assess known protein-protein interactions. Although the STRING database has information related to known and predicted protein-protein interactions, only known interactions were selected for this analysis, for accuracy. Figure 1 shows the STRING network of interactions for the top-ranked genes. The network view summarizes the associations for the group of gene products. The network edges represent the predicted functional associations and each colour represents a different line of evidence. For the genes that were found to be linked through protein-protein interaction, the source of evidence for the interactions and confidence scores are summarized in Table 3. Significantly fewer protein-protein interactions were observed within the low-ranked gene list.

**Table 3:** Known protein-protein interaction for the prioritized candidate genes obtained using STRING

		**Confidence Scores**		**Combined confidence score**
**Gene 1**	**Gene 2**	**Experimental**	**Knowledge**	
*TGFB1*^1,2^	*DCN*^2^	0.747	0.8	0.949
*BRCA1*	*TP53*^1^	0.935	0	0.935
*CTNNB1*	*CREBBP*^2^	0.912	0.9	0.991
*MADH2*^2^	*CREBBP*^2^	0.937	0	0.937
*BCL2L1*	*TP53*^1^	0.935	0	0.935
*CTNNB1*	*PITX2*^2^	0	0.9	0.9
*PPARBP*	*TP53*^1^	0.935	0	0.935
*CASP3*^1^	*RB1*	0.747	0.9	0.974
*BMI1*	*PHC2*	0.997	0	0.997
*HMGB1*	*TP53*^1^	0.935	0	0.935
*LDB1*	*LMO4*	0.938	0	0.938
*MADH2*^2^	*TGFB2*^1.2^	0	0.9	0.9
*EGFR*^1^	*MAPK3*^1,2^	0	0.9	0.9
*BRCA1*	*RB1*	0.935	0	0.935
*PITX2*^2^	*LDB1*	0	0.9	0.9
*RXRA*	*CREBBP*^2^	0.03	0.9	0.903
*TP53*^1^	*CREBBP*^2^	0.983	0.9	0.998
*CTNNB1*	*GJA1*	0	0.9	0.9
*PITX2*^2^	*CREBBP*^2^	0	0.9	0.9
*VEGF*	*ARNT*	0	0.9	0.9

The available evidence for the most significant interactions as well as the confidence score assigned for the interactions are shown

^1 ^Members of/linked to the MAPK signalling pathway

^2 ^Members of/linked to the TGF-β signalling pathway

^3^Members of/linked to the Hedgehog signalling pathway

#### Functional enrichment analysis using DAVID

DAVID (Database for annotation, visualization and integrated discovery) [36] was used to assess functional enrichment within the top-ranked candidate gene list. Firstly, the analysis focusing on pathway maps highlighted a number of pathways significantly represented within the gene list, with the transforming growth factor (TGF-β) signalling pathway being most over-represented within the list (Table 4). This enrichment was not observed on the low-ranked gene list (data not shown). Furthermore, significant enrichment of Gene Ontology (GO) terms was observed in the top-ranked list for all three of the GO – categories. The GO terms found to be significantly enriched for the top-ranked gene list are shown in Additional file 2.

**Table 4:** Biological pathways significantly over-represented among the top-ranked candidate genes

**Pathway**	**Gene Count**	***P*-value^1^**	***P*-value^2^**
TGF-b signaling pathway	9	0.0000067	0.00001
Hedgehog signaling pathway	7	0.000038	0.000036
MAPK signaling pathway	13	0.000078	0.0006
Adherens junction	7	0.00035	0.00051
Cell cycle	8	0.00036	0.0012
Neurodegenerative disorders	5	0.00075	0.0023
Regulation of actin cytoskeletan	9	0.0035	0.014
Focal adhesion	9	0.004	0.022
Gap junction	6	0.0059	0.0079
Cytokine-cytokine receptor interaction	9	0.011	0.0017
Epithelial cell signaling in H. Pylori infection	4	0.018	0.029

The gene count indicates how many genes from a particular pathway were present in the candidate gene list of 87 genes. Note that varying *P*-values were obtained depending on the background list used

^1^*P*-value obtained using the Homo sapiens gene list as a background list to the top-ranked candidate genes

^2^*P*-value obtained using the original candidate gene list as a background list to the top-ranked candidate genes

#### Promoter element binding site analysis

As shown in Tables 5 and 6, the promoter analysis detected 15 transcription factors (TF) that appear in promoter elements (PE) or pairs of PE that are significantly statistically enriched in the target promoter set as opposed to the background set. The conditions for selection PE of Tables 5 and 6 were that PE (or their combination) has to appear in at least 5% of promoters in the target set and to have over-representation index (ORI) (see Bajic *et al*. [37]) of at least 2. These are AP-2, C/EBP, E2F, ETF, LEF1, MAZ, MAZR, MZF1, Pax-4, Sp1, Spz1, TATA, TFII-I, VDR, ZF5. In Tables 5 and 6, PE or their combinations that have been found in significantly enriched proportions relative to the background promoter set, are denoted by a + sign in the column of the over-representation index (ORI). Further analysis suggests that TF that potentially bind these transcription factor binding sites (TFBS), are part of the group of TF that are dominant transcriptional regulators of our promoter target set (Tables 5 and 6). Results from the promoter element binding site analysis are shown in Additional file 3.

**Table 5:** Promoter elements found to be enriched in the target promoter set relative to the background promoter set

**Promoter elements**	**ORI**	**TAR (%)**	**BCG (%)**	**Probability of finding PE in target set**	**Probability of finding PE in background set**	**TAR (n)**	**BCG (n)**	**TAR Total**	**BCG Total**	***P*-value**
-1 MAZR	11.5685	5.7	2.07	0.00009	0.00002	31	212	544	10255	0.002
+1 MAZR	5.6322	5.15	2.08	0.00005	0.00002	28	213	544	10255	0.033
-1 TATA	2.9231	16.36	9.76	0.00017	0.00010	89	1001	544	10255	0.002
-1 TFII-I	2.8865	18.2	10	0.00017	0.00011	99	1025	544	10255	<0.001
-1 MAZ	2.6342	29.96	20.03	0.00040	0.00022	163	2054	544	10255	<0.001

The criteria for selecting PE as enriched was that it has to appear in at least 5% of the target promoter sequences and to have over-representation index (ORI) of at least 2. PE that appear in statistically significant proportion in the target set are denoted by + in the ORI column.

**Table 6:** Pairs of promoter elements found to be enriched in the target promoter set relative to the background promoter set

**Pairs of promoter elements**	**ORI**	**TAR (%)**	**BCG (%)**	**Probability of finding PE in target set**	**Probability of finding PE in background set**	**TAR (n)**	**BCG (n)**	**TAR Total**	**BCG Total**	***P*-value**
-1 MZF1/+1 E2F	17.1036	6.62	1.47	0.00007	0.00002	36	151	544	10255	0.000003
-1 LEF1/-1 Pax-4	14.6726	6.62	1.77	0.00007	0.00002	36	182	544	10255	0.000254
-1 C/EBP/+1 VDR	10.1454	6.43	1.85	0.00006	0.00002	35	190	544	10255	0.002385
+1 C/EBP/+1 VDR	9.1022	6.99	2.42	0.00009	0.00003	38	248	544	10255	0.041880
-1 MAZ/-1 VDR	9.0220	9.19	2.89	0.00015	0.00005	50	296	544	10255	0.000012
-1 MZF1/-1 MZF1	8.4560	5.51	1.6	0.00007	0.00003	30	164	544	10255	0.039710
-1 ETF/-1 VDR	7.6725	7.54	2.68	0.00011	0.00004	41	275	544	10255	0.023710
-1 AP-2/-1 ETF	6.8853	12.87	5.11	0.00023	0.00009	70	524	544	10255	0.000015
-1 MAZ/+1 Sp1	6.4534	9.56	2.63	0.00012	0.00007	52	270	544	10255	0.000000
-1 Spz1/-1 Spz1	6.0515	14.34	5.57	0.00021	0.00009	78	571	544	10255	<0.00001
-1 VDR/-1 Spz1	5.4345	14.34	6.54	0.00024	0.00010	78	671	544	10255	0.000429
-1 ETF/-1 E2F	5.4001	11.76	5.22	0.00020	0.00009	64	535	544	10255	0.007227
-1 Spz1/-1 VDR	5.2463	13.6	6.58	0.00025	0.00010	74	675	544	10255	0.013100
-1 VDR/+1 ZF5	4.4308	14.15	6.59	0.00018	0.00009	77	676	544	10255	0.001322

The criteria for selecting pairs of PE as enriched was that it has to appear in at least 5% of the target promoter sequences and to have over-representation index (ORI) of at least 2. PE that appear in statistically significant proportion in the target set are denoted by + in the ORI column.

## Discussion

Our challenge was to select a highly likely group of candidate genes for susceptibility to FAS, in the absence of genetic linkage evidence. In this paper a computational approach to candidate disease gene identification is proposed as an effective first line of candidate gene identification for a complex disease such as FAS. Mining of gene expression data was used to generate an extensive list of candidate genes which were compared to filtered criteria specific gene lists using 29 criteria to select the most likely candidate genes. The prioritization method described here is based on a computational model of a researcher's approach to selecting candidate genes, i.e. based on published information; but may also select non-intuitive candidate genes. In summary, various relevant database sources are accessed to establish whether a candidate gene and its product exhibit the biological characteristics consistent with that particular disease.

### Candidate gene selection and -prioritization

A method that employs an integrative literature- and data mining approach to select candidate genes was used to select candidate genes for FAS [31]. This method extracted a gene list of 10174 genes. This list is relatively unspecific, and is likely to have a high false-positive rate. The most plausible explanation for the selection of such a large, ambiguous list is a lack of detailed information about the source of cDNA libraries, with the result that more general terms from higher up the ontology hierarchy are often used for annotation of the gene. This prompted us to devise a prioritization method to rank genes from the candidate gene list using many different data sources for laboratory investigation of individual candidate genes. A binary evaluation method was used to rank the candidate genes in the list, facilitating the selection of 87 top-ranked genes as the most likely candidate genes for further investigation.

Further analysis with available online tools such as DAVID and STRING highlighted protein-protein interaction, functional enrichment and probable biological significance among the top-ranked genes. STRING was used to investigate protein-protein interaction among the prioritized candidate genes, and highlighted a group of genes that interact (Figure 1 and Table 3). The candidate gene selection method described here focuses on gene annotation, and it is therefore possible that the top ranking genes are better annotated than low-ranked genes. Therefore the absence of protein-protein interaction among the low-ranked genes is not necessarily a reflection on level of interaction but may be related to the level of understanding of the gene and its function. It is accepted that the genes underlying complex disease (such as FAS) will be plentiful and the interactions between these factors are likely to be intricate. For this reason, STRING is a useful tool to highlight genes within the top-ranked gene list that interact and that may have a cooperative effect on disease outcome.

DAVID elucidates functional enrichment and biological significance within the top-ranked gene list, and highlighted the TGF-β and Mitogen-Activated Protein Kinase (MAPK) signalling pathways as primary candidate pathways for FAS development.

As a way of further assessing the list of 87 prioritized FAS candidate genes they were cross-matched against candidate genes for alcoholism, obtained using Convergent Functional Genomics [32]. Although the two phenotypes are very different, one would expect some overlap in prioritized candidate genes since many of the mothers of FAS children suffer from alcoholism. The two prioritized candidate gene lists (87 genes for FAS and 65 for alcoholism) had only two high priority candidate genes in common – GNAS complex locus (*GNAS*) and high mobility group protein B1 (*HMGB1*). The remaining 63 candidate genes for alcoholism were also present in the initially selected list of 10174 genes, but were ranked below the arbitrary cut-off of 11/29 criteria used to select the highly prioritized candidate list for FAS.

Incorporating the set of alcoholism genes as a selection criterion into the binary evaluation method only added two more genes to the prioritized list. These were G1/S-specific cyclin-D1 (*CCND1*) and insulin-like growth factor I receptor (*IGF1R*). Both gene products contribute to cell proliferation and differentiation, and exhibit characteristics that also make them likely candidate genes for FAS. However, neither directly interacts in the two main prioritized pathways (TGF-β or MAPK signalling pathway). This comparison shows that these two related diseases (due to the involvement of alcohol in both) have potentially common genetic factors, but that they also exhibit diversity in terms of genetic susceptibility. This gene list was therefore not included in the final binary filtering analysis.

### Prioritized pathways – relevance to FAS development

#### TGF-β signalling pathway

FAS is a complex disease, suggesting that the genetic factors underlying susceptibility to FAS may be plentiful and the interactions between these factors, as well as environmental factors are likely to be intricate. The computational approach described here highlights genes that are important players in various signalling pathways, in particular the TGF-β and MAPK pathways. These genes play pivotal roles during embryogenesis and development (Table 2) and have a potential role in the distinct characteristics associated with FAS, i.e. CNS dysfunction, craniofacial abnormalities and growth retardation. CNS dysfunction is the most severe and permanent consequence of *in utero *alcohol exposure and the only feature present in all other disorders in FASD. These observations make the TGF-β signalling pathway an interesting focus point, as it is essential in both fetal development and also CNS development [38].

TGF-β signalling controls a diverse array of cellular processes, including cell proliferation and apoptosis, cell differentiation and specification of cellular phenotypes and developmental fate [39]. TGF-β is also important in neuronal migration and axonal growth, and regulates the formation of various craniofacial structures [40,41].

Early exposure to ethanol inhibits such TGF-β regulated processes as cortical cell proliferation and neuronal migration, disrupts axonal growth and up-regulates cell adhesion molecule expression [40]. It can therefore be suggested that members of the TGF-β signalling pathway interact with ethanol, and/or its metabolic breakdown products, and that ethanol may have a detrimental effect on the efficiency of this developmentally essential pathway. Investigating the role of TGF-β components present among the top-ranked genes may clarify part of the genetic component contributing to susceptibility for FAS development.

The hypothesis that TGF-β signalling pathway genes may be involved in FAS susceptibility is even more compelling when considering the major role of this pathway in neuronal apoptosis. Several studies have shown that alcohol suppresses neuronal activity, resulting in a pro-apoptotic environment in the developing brain [42-44]. Alcohol-induced neural apoptosis has been observed throughout the developing CNS, including all levels of the spinal cord, brain stem, cerebellum, midbrain and forebrain. Furthermore, alcohol has been observed to diminish neurons from various parts of the developing visual-, auditory- and memory systems of the developing brain [43]. This pro-apoptotic effect of alcohol provides a probable explanation for the long-term CNS dysfunction and diminished brain size associated with FAS, and could be mediated by the TGF-β pathway. Alcohol has an array of molecular pathway targets and modes of inducing apoptosis and the candidate disease genes selected using this method have a strong role to play in apoptosis.

Genetic mutations in members of the TGF-β signal pathway, generally result in tumorigenesis, and have been repeatedly linked to human cancer [46-49] TGF-β dysfunction is also causal for hereditary hemorrhagic telangiectasia [50], corneal dystrophy [51], Camurati-Engelmann Disease of bone [52] glomerulonephritis [53], scar formation [54], keloids [55], pulmonary fibrosis [56], and liver cirrhosis [57]. Recent studies also propose a role for TGF-β signalling in Alzheimer's disease pathology [58,59]. However, no such link has to date been proposed between genetic susceptibility to FAS development and disruption of the TGF-β pathway. Given the above-mentioned experimental evidence, the TGF-β pathway, and specifically its components that were top-ranked using this computational approach, is an attractive focus for a genetic association study.

#### MAPK signalling pathway

The MAPK pathway transmits a large variety of external signals, leading to a wide range of cellular responses, including growth, differentiation, inflammation and apoptosis [60]. This pathway is very complex and includes many protein components. MAPK-pathway components have been shown to be involved in both the initiation and regulation of meiosis, mitosis, and post-mitotic functions, and in cell differentiation by phosphorylating a number of transcription factors [61].

The MAPK signalling pathway can be activated by a variety of stimuli, including growth factors, cytokines and differentiation factors [60] as well as external stress factors, such as alcohol [62]. Recent studies have investigated the effect of controlling second-messenger signalling on neuronal migration in a mouse model of FAS [63]. It was shown that experimental manipulation of these second-messenger pathways, through stimulating calcium- and cGMP signalling or inhibiting cAMP signalling, completely reversed the action of ethanol on neuronal migration *in vitro *as well as *in vivo*. Each investigated second messenger had multiple but distinct downstream targets, including MAPK.

#### Hedgehog signalling pathway

The hedgehog signalling pathway also received a highly significant ranking among the pathways identified to be enriched within the candidate list. The hedgehog signalling pathway is a key regulator of embryonic development and is highly conserved. Knock-out mouse models lacking components of this pathway have been observed to develop malformations in the CNS, musculoskeletal system, gastrointestinal tract and lungs [64].

FAS animal models portray a strikingly similar craniofacial phenotype to mouse models treated with antibodies that block Hedgehog signalling components, specifically the sonic hedgehog (Shh) molecule [65-67] Further studies to expose the role of Shh in fetal alcohol syndrome, showed that alcohol resulted in a significant decrease in Shh levels in the developing embryo, as well as a decrease in the level of other transcripts involved in Shh signalling. Furthermore it was observed that the addition of Shh after ethanol treatment led to fewer apoptotic cranial neural crest cells, resulting in a significant decrease in craniofacial anomalies [68]. These results give compelling support that the components of the Hedgehog signalling pathway may also be important in the genetics of FAS.

### Transcriptional regulators of the prioritised genes

All TFBS that are found to be statistically significant for FAS are known to be involved in gene expression and regulation in the CNS, endocrine system or development. The AP-2 family of TF is crucial for neural gene expression and neuronal development [69]; C/EBP is involved in neuronal signalling [70]; the E2F family of TF is one of the key controllers of cell-cycle and has a known role in pathways controlling neuron death [71]; ETF, the epidermal growth factor receptor-specific TF, is implicated in neuroblastoma [72]; LEF1 is expressed in the nerve system of mammals [73]; MAZ is involved in Hodgkin's disease and paraneoplastic cerebellar dysfunction [74] and during neuronal differentiation [75]; MAZR is implicated in the development of mouse limb buds [76]; MZF1 is involved in development [77] and implicated in the control of the BACE1 gene related to Alzheimer's disease [78]; Pax-4 is involved in the endocrine system and development [79]; Sp1 has multiple roles, but, for example, controls expression of Na+,K+-ATPase in neuronal cells [80]; Spz1 is involved in cell-proliferation [81]; TATA binding proteins are implicated in various processes involved in brain [82]; the TFII-I transcription factor family is implicated in craniofacial development of humans and mice [83]; VDR is associated with increased risk of schizophrenia [84]; and ZF5 is implicated in neuroblastoma differentiation [85]. These results support the prioritization of biologically relevant candidate disease genes.

## Conclusion

The results obtained in this study suggest that making a clinically-informed selection from the evidence obtained from literature- and database-mining is an effective approach for candidate disease gene selection and -prioritization. The main limitation of this approach is that it is primarily based on gene annotation, and that it is therefore biased towards selecting better annotated genes. Furthermore, some clinical understanding of the disease aetiology is needed to aid the clinically-informed binary evaluation, and this process could be partly subjective and researcher-specific. The effectiveness of this approach critically depends on the disease under investigation being clearly defined both molecularly and physiologically, in order to avoid erroneous associations. A multitude of biological processes are affected by the insult of alcohol exposure, particularly given a predisposing genetic background. FAS as a developmental disorder represents with a spectrum of structural, behavioral and neurocognitive disabilities, which complicates this process of clearly defining focus. This is evident when considering the ambiguous results obtained when using the method that only considers general anatomical terms to select candidate genes [31]. This encouraged the inclusion of the binary prioritization technique to further enhance the selection process.

A further limitation of employing this approach in selecting candidate genes for a developmental disorder lies in the limited knowledge available regarding the mechanisms involved in such a disorder. The developing organism undergoes many rounds of pattern formation, generating complexity with each ensuing round of cell division and with cell differentiation. Even though the pathways identified using this technique are general fundamental role players in embryogenesis and development, the technique allowed the focus to fall on specific candidate genes within these pathways for investigation.

The computational approach described here has been used to select and refine a 'most likely' candidate gene list according to known characteristics of FAS. We have demonstrated that we can identify likely candidates that are biologically relevant to the disease, and therefore appropriate for gene association studies. By refining the candidate gene list for FAS using a binary evaluation approach, we selected a subset of biologically relevant candidate genes for experimental validation.

## Methods

### Literature search

Abstracts related to FAS were obtained from the PubMed scientific literature database. In order to obtain all relevant literature, PubMed's automatic term mapping search of the literature might not be sufficient and a more robust search option of using Medical Subject Heading (MeSH) terms was implemented. Using this option also implies that all equivalent synonyms or lexical variants in English will be included in the search [86]. Literature related to FAS was obtained using the following query: "(fetal alcohol syndrome [MH]) OR (fetal alcohol spectrum disorder* [tw])" Limits: only items with abstracts, English.

### Literature mining

The online literature mining tool DDE [87] was used to extract eVOC ontology terms from the body of literature. The eVOC ontology is a controlled vocabulary used to describe the sample source of cDNA and SAGE libraries and labelled target cDNAs for microarray experiments. eVOC contains four major orthogonal ontologies – anatomical system, cell type, pathology and developmental stage [88]. DDE provides summarized information from a body of submitted PubMed abstracts about frequency of occurrence of ontology terms within the text. This assists biologists in uncovering possible functional associations between disease and gene expression site. Following the method of Tiffin *et al*. [31], only eVOC anatomy terms were used to extract the initial candidate gene list. Cell type terms were used to populate criteria lists for the binary filtering approach (Table 1). Terms extracted matching to the developmental- and pathology ontologies were uninformative in this case (terms such as pathology or adult were extracted) and it was deemed that populating criteria lists using these terms would not contribute positively to the selectivity of the binary evaluation system. Therefore these terms were not further included.

### Candidate gene selection

**Figure 2:**
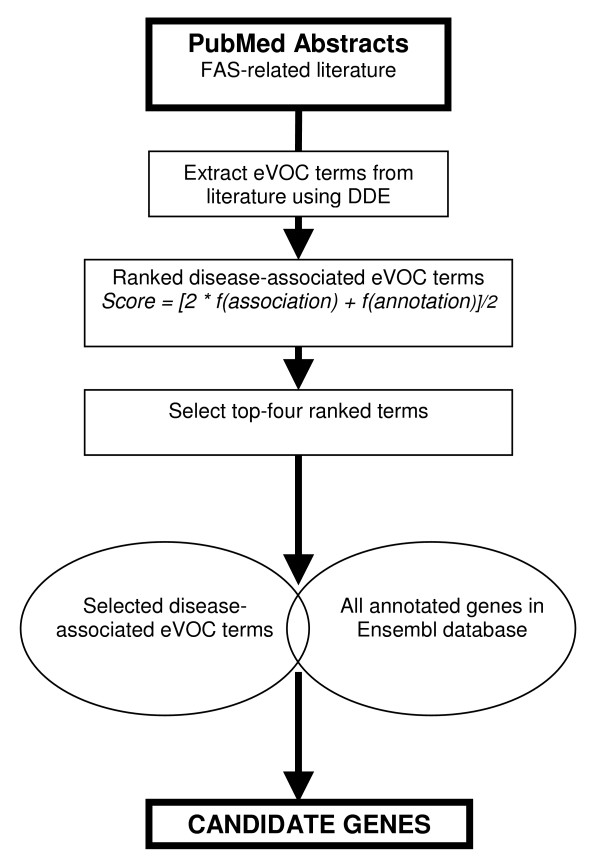
The method of integrated literature- and data mining to identify an initial list of putative candidate genes.

The method previously described by Tiffin *et al*. [31] was used to extract candidate genes based on the information obtained from the literature mining. Figure 2 illustrates the process of literature- and data-mining used to select candidate genes. Briefly, this method ranks the extracted eVOC terms by calculating a ranking score for each associated eVOC term, according to the frequency of association and the frequency of annotation of the eVOC term. The four top-scoring eVOC terms were selected from the ranked list, and compared with eVOC terms annotated to genes within the Ensembl database (Ensembl v33, September 2005) to select candidates. The system allows for one mismatch, such that candidates selected are those annotated with at least three of the four top-scoring eVOC terms. This approach was tested by the authors on a subset of genes representative of those that might be selected by a linkage analysis study, and not the full complement of genes in the Ensembl database, as in the current study.

### Binary filtering and prioritization of candidate genes

The integrated literature- and data-mining approach to identify candidate genes focuses exclusively on anatomical sites related to the disease of interest, and results in a large list of genes. In order to obtain a more focused assessment of the most likely candidates from this gene list, other criteria pertinent to FAS were investigated. Five main categories of criteria were used – cell type, biological process, homology, imprinted genes and phenotype simile. For each category there are multiple gene lists, each specified by one criterion (Table 1). The criteria-specific gene lists generated were compared to the candidate gene list (obtained from the integrated data- and literature-mining approach described above) to create a binary matrix. The binary evaluation was performed as follows: A gene in the candidate gene list was assigned a 1 when that gene was also present in the gene list obtained by a specific criterion. If the gene was absent from that list it was assigned a 0.

For each of the genes we calculated the final binary score, simply by summing all binary scores for each of the criteria used. Then we ranked all genes based on this score, with those having higher scores being higher in the rank list. Genes in the candidate list that were present in most criteria lists (i.e. those genes that obtaining the most 1-scores in the binary matrix) received the highest rank as candidates. This follows the premise that genes most commonly selected from additional independent sources possess characteristics that make them more promising candidates. Similarly, genes that were selected by only one or none of the additional criteria have a lower rank and are considered to be weak candidates.

A description of each category of criterion and the information used to assess the criteria are given below:

#### Cell Type

DDE was used to extract all eVOC cell type terms from the disease-related literature. Cell type ontology terms found to be associated with FAS were compared with eVOC terms annotated to genes within the Ensembl database to select a list of genes.

#### Biological Process

Disease-related literature contains terms describing functional aspects related to the disease. Dragon TF Association Miner (DTFAM) is an online tool for text-mining of PubMed abstracts to discover potential functional association of GO-terms and diseases [89]. DTFAM was used to extract all GO terms from the abstracts of disease-related literature. Of the terms extracted, terms falling in the molecular function (binding) and cellular component (membrane, nucleus, chromosome and intracellular) ontologies were not included in the analysis, as we considered these terms non-specific with regard to FAS and non-specific in general. Terms from the biological process ontology considered uninformative were also eliminated. This includes terms such as pathogenesis or lactation that would appear in the relevant literature due to subject matter described, and not because of relevance to disease. Genes annotated with the selected GO terms extracted from the literature were obtained from the Ensembl database, and each individually used to populate a criteria list.

#### Animal model homology

Animal models offer major contributions to the understanding of human disease. Although many different animal models for FAS have been developed [90], the mouse model seems to correlate best to the effects of prenatal alcohol exposure observed in humans [91]. The Mouse Genome Database (MGD) documents the mouse as a model system for human biology and disease process research [92]. MGD integrates genetic and genomic data for the mouse, including sequence sets, mapping details, GO annotations, allele descriptions and mutant phenotype characteristics. Furthermore MGD provides a curated set of mammalian orthologues [93].

Human orthologues to the following categories of mouse genes were selected:

▪ Genes associated with phenotypes affected by prenatal alcohol exposure

▪ Genes expressed at different developmental stages

▪ Genes expressed in the developing brain

#### Phenotype simile

It is assumed that similar phenotypes may be influenced by similar genotypes [94]. The main characteristics of FAS are growth retardation, distinct craniofacial dysmorphology and CNS dysfunction. The neurodevelopmental consequences of CNS dysfunction due to prenatal alcohol exposure include cognitive deficits (often mental retardation), executive functioning deficits, motor functioning delays and problems with attention, hyperactivity and social skills [95]. Terms describing key phenotypes associated with FAS (mental retardation, microcephaly, craniofacial, hyperactivity and growth retardation) were used to search for genes in the Gene Cards catalogue [96]. Genes linked to these phenotype terms were used to create the criteria lists.

#### Imprinted Genes

Genomic imprinting refers to an epigenetic modification, resulting in the control of gene expression as dictated by parental inheritance [97]. One of the well-known features of imprinted genes is differential allele-specific DNA methylation, and is usually found in regions known as differentially methylated regions. Differentially methylated regions include imprinting control regions, and it is thought that all clusters of imprinted genes have imprinting control regions, which are differentially methylated [98]. The expression of many prokaryotic and eukaryotic genes is regulated through the methylation of DNA [99]. Animal studies have shown that *in utero *ethanol exposure inhibits fetal DNA methylation [100,101]. Since DNA methylation and imprinting play an important role in the regulation of gene expression during embryogenesis [101,102]. and consequent development, ethanol-associated alterations in fetal DNA methylation may contribute to the developmental abnormalities seen in FAS. One of the criteria gene lists therefore contained all known imprinted genes, obtained from the imprinted gene catalogue [103] and the imprinted gene database [104].

### Evaluation of biological significance of prioritized genes

Protein-protein interactions, functional enrichment and common promoter element binding sites were investigated for the top-ranked genes (i.e. those with the highest binary score) to assess their biological significance as candidates for FAS. In comparison, the lowest-ranked genes were similarly evaluated to assess the validity of the ranking system in selecting biologically relevant genes from the original candidate gene list.

#### Protein-protein interactions

Understanding interactions between proteins involved in common cellular functions may indicate how such interactions can influence disease outcome. Protein-protein interactions were analysed using data contained in the STRING database [35]. The STRING database provides a comprehensive source of protein-protein association evidence under a common framework. STRING integrates protein-protein interaction data from both experimental evidence databases (such as BIND, DIP and MINT) as well as inferred protein-protein interactions obtained by using *de novo *prediction tools (such as Predictome), or functional grouping databases (such as Reactome or KEGG). The user can select which lines of evidence to use, and each predicted association in the database is assigned a confidence score, based on comparison to a common reference set of true associations. The top-ranked candidate genes were used as input, and protein-protein interactions based on experimental evidence, and functional groupings were selected as evidence. A high confidence score for evidence was selected (90%).

#### Functional enrichment analysis using DAVID

DAVID is an online tool that integrates genomic functional annotations to reveal biologically relevant enrichment in a gene list [36]. DAVID promotes functional discovery through exploration of biochemical pathway maps, functional classification using GO terms and conserved protein domain architecture. Data from various sources are integrated into DAVID, including GenBank, UniGene, RefSeq, Locuslink, KEGG, OMIM and GO. The top-ranked genes were submitted as a list, which was then compared to a background gene list to assess functional enrichment within the list. The background list can either be all genes in the human genome, or a sub-set of genes. Two analyses were performed – firstly with the original candidate gene list of 10174 genes as background, and secondly using the *Homo sapiens *default background list from the DAVID website as background.

#### Promoter element binding site analysis

To investigate potential drivers of transcription initiation of the top-ranked candidate genes and associate the prioritized genes better to the FAS phenotype, mammalian TFBS were predicted. This was done using matrix models in Transfac database v9.4 for the promoters of all prioritized genes. Thresholds that correspond to the minimum number of false positive predictions as defined by minFP profiles in Transfac were used. The same process was applied to 10255 human promoters according to Bajic *et al*.[105]. Using the methodology of contrasting target promoter set with the background set of 10255 human promoters [37], the most dominant promoter elements were determined. A promoter element is defined as a TFBS and the strand where it is predicted, or as a pair of these if they are at the maximum distance of 50 nucleotides.

## Authors' contributions

ZL performed the literature mining and candidate gene selection using scripts written by NT, binary filtering and prioritization of candidate genes, and protein-protein interaction and functional enrichment analysis; wrote some basic programs for database mining, assembled the set of candidate genes; contributed to the study design, and drafted the manuscript. NT was involved in the initial stages of the project and assisted in literature mining and selection of the initial candidate gene set, and participated in manuscript preparation. OH and VBB performed the transcription factor binding site analysis, and participated in manuscript preparation. MR and WH conceived of this study, and participated in its design and coordination, and participated in manuscript preparation. All authors read and approved the final manuscript.

## Supplementary Material

Additional file 1 The 87 top-ranked genes for FAS identified using binary matrix filtering Click here for file

Additional file 2 GO term annotations significantly over-represented among the top-ranked genes. The table provided represent the GO biological process, cellular component and molecular function terms over-represented among the top-ranked genes Click here for file

Additional file 3 Promoter element binding site analysis. The tables provided represent the promoter elements that have been found in the target promoter set relative to the background promoter set (Table 1) and the pairs of promoter elements at maximum mutual distance of 50 nucleotides (Table 2) that have been found in the target promoter set relative to the background promoter set. Click here for file

## References

[B1] May PA, Gossage JP, Marais AS, Adnams CM, Hoyme HE, Jones KL, Robinson LK, Khaole NC, Snell C, Kalberg WO, Hendricks L, Brooke L, Stellavato C, Viljoen DL (2007). The epidemiology of fetal alcohol syndrome and partial FAS in a South African community. Drug Alcohol Depend.

[B2] Viljoen DL, Gossage JP, Brooke L, Adnams CM, Jones KL, Robinson LK, Hoyme HE, Snell C, Khaole NC, Kodituwakku P, Asante KO, Findlay R, Quinton B, Marais AS, Kalberg WO, May PA (2005). Fetal alcohol syndrome epidemiology in a South African community: a second study of a very high prevalence area. J Stud Alcohol.

[B3] Abel EL (1995). An update on incidence of FAS: FAS is not an equal opportunity birth defect. Neurotoxicol Teratol.

[B4] Barr HM, Streissguth AP (2001). Identifying maternal self-reported alcohol use associated with fetal alcohol spectrum disorders. Alcohol Clin Exp Res.

[B5] Clarren SK, Alvord EC, Sumi SM, Streissguth AP, Smith DW (1978). Brain malformations related to prenatal exposure to ethanol. J Pediatr.

[B6] Sulik KK, Johnston MC (1983). Sequence of developmental alterations following acute ethanol exposure in mice: craniofacial features of the fetal alcohol syndrome. Am J Anat.

[B7] Day NL, Zuo Y, Richardson GA, Goldschmidt L, Larkby CA, Cornelius MD (1999). Prenatal alcohol use and offspring size at 10 years of age. Alcohol Clin Exp Res.

[B8] Sampson PD, Streissguth AP, Bookstein FL, Little RE, Clarren SK, Dehaene P, Hanson JW, Graham JM (1997). Incidence of fetal alcohol syndrome and prevalence of alcohol-related neurodevelopmental disorder. Teratology.

[B9] Chaudhuri JD (2000). Alcohol and the developing fetus – a review. Med Sci Monit.

[B10] Streissguth AP, Dehaene P (1993). Fetal alcohol syndrome in twins of alcoholic mothers: concordance of diagnosis and IQ. Am J Med Genet.

[B11] Michelson P, Hartwig C, Schachner M, Gal A, Veske A, Finckh U (2002). Missense mutations in the extracellular domain of the human neural cell adhesion molecule L1 reduce neurite outgrowth of murine cerebellar neurons. Hum Mutat.

[B12] Thomas JD, Burchette TL, Dominguez HD, Riley EP (2000). Neonatal alcohol exposure produces more severe motor coordination deficits in high alcohol sensitive rats compared to low alcohol sensitive rats. Alcohol.

[B13] Ogawa T, Kuwagata M, Ruiz J, Zhou FC (2005). Differential teratogenic effect of alcohol on embryonic development between C57BL/6 and DBA/2 mice: a new view. Alcohol Clin Exp Res.

[B14] Boehm SL, Lundahl KR, Caldwell J, Gilliam DM (1997). Ethanol teratogenesis in the C57BL/6J, DBA/2J, and A/J inbred mouse strains. Alcohol.

[B15] Gilliam DM, Mantle MA, Barkhausen DA, Tweden DR (1997). Effects of acute prenatal ethanol administration in a reciprocal cross of C57BL/6J and short-sleep mice: maternal effects and nonmaternal factors. Alcohol Clin Exp Res.

[B16] McCarthy MI, Smedley D, Hide W (2003). New methods for finding disease-susceptibility genes: impact and potential. Genome Biol.

[B17] Stoler JM, Ryan LM, Holmes LB (2002). Alcohol dehydrogenase 2 genotypes, maternal alcohol use, and infant outcome. J Pediatr.

[B18] McCarver DG, Thomasson HR, Martier SS, Sokol RJ, Li T (1997). Alcohol dehydrogenase-2*3 allele protects against alcohol-related birth defects among African Americans. J Pharmacol Exp Ther.

[B19] Jacobson SW, Carr LG, Croxford J, Sokol RJ, Li TK, Jacobson JL (2006). Protective effects of the alcohol dehydrogenase-ADH1B allele in children exposed to alcohol during pregnancy. J Pediatr.

[B20] Viljoen DL, Carr LG, Foroud TM, Brooke L, Ramsay M, Li TK (2001). Alcohol dehydrogenase-2*2 allele is associated with decreased prevalence of fetal alcohol syndrome in the mixed-ancestry population of the Western Cape Province, South Africa. Alcohol Clin Exp Res.

[B21] George RA, Liu JY, Feng LL, Bryson-Richardson RJ, Fatkin D, Wouters MA (2006). Analysis of protein sequence and interaction data for candidate disease gene prediction. Nucleic Acids Res.

[B22] Aerts S, Lambrechts D, Maity S, Van Loo P, Coessens B, De Smet F, Tranchevent LC, De Moor B, Marynen P, Hassan B, Carmeliet P, Moreau Y (2006). Gene prioritization through genomic data fusion. Nat Biotechnol.

[B23] Franke L, Bakel H, Fokkens L, de Jong ED, Egmont-Petersen M, Wijmenga C (2006). Reconstruction of a functional human gene network, with an application for prioritizing positional candidate genes. Am J Hum Genet.

[B24] Adie EA, Adams RR, Evans KL, Porteous DJ, Pickard BS (2006). SUSPECTS: enabling fast and effective prioritization of positional candidates. Bioinformatics.

[B25] Freudenberg J, Propping P (2002). A similarity-based method for genome-wide prediction of disease-relevant human genes. Bioinformatics.

[B26] Kent WJ, Hsu F, Karolchik D, Kuhn RM, Clawson H, Trumbower H, Haussler D (2005). Exploring relationships and mining data with the UCSC Gene Sorter. Genome Res.

[B27] Lopez-Bigas N, Ouzounis CA (2004). Genome-wide identification of genes likely to be involved in human genetic disease. Nucleic Acids Res.

[B28] Perez-Iratxeta C, Wjst M, Bork P, Andrade MA (2005). G2D: a tool for mining genes associated with disease. BMC Genet.

[B29] Turner FS, Clutterbuck DR, Semple CA (2003). POCUS: mining genomic sequence annotation to predict disease genes. Genome Biol.

[B30] van Driel MA, Cuelenaere K, Kemmeren PP, Leunissen JA, Brunner HG, Vriend G (2005). GeneSeeker: extraction and integration of human disease-related information from web-based genetic databases. Nucleic Acids Res.

[B31] Tiffin N, Kelso JF, Powell AR, Pan H, Bajic VB, Hide WA (2005). Integration of text- and data-mining using ontologies successfully selects disease gene candidates. Nucleic Acids Res.

[B32] Rodd ZA, Bertsch BA, Strother WN, Le-Niculescu H, Balaraman Y, Hayden E, Jerome RE, Lumeng L, Nurnberger JI, Edenberg HJ, McBride WJ, Niculescu AB (2007). Candidate genes, pathways and mechanisms for alcoholism: an expanded convergent functional genomics approach. Pharmacogenomics J.

[B33] Bertsch B, Ogden CA, Sidhu K, Le-Niculescu H, Kuczenski R, Niculescu AB (2005). Convergent functional genomics: a Bayesian candidate gene identification approach for complex disorders. Methods.

[B34] Tiffin N, Adie E, Turner F, Brunner HG, van Driel MA, Oti M, Lopez-Bigas N, Ouzounis C, Perez-Iratxeta C, Andrade-Navarro MA, Adeyemo A, Patti ME, Semple CA, Hide W (2006). Computational disease gene identification: a concert of methods prioritizes type 2 diabetes and obesity candidate genes. Nucleic Acids Res.

[B35] von Mering C, Jensen LJ, Snel B, Hooper SD, Krupp M, Foglierini M, Jouffre N, Huynen MA, Bork P (2005). STRING: known and predicted protein-protein associations, integrated and transferred across organisms. Nucleic Acids Res.

[B36] Dennis G, Sherman BT, Hosack DA, Yang J, Gao W, Lane HC, Lempicki RA (2003). DAVID: Database for Annotation, Visualization, and Integrated Discovery. Genome Biol.

[B37] Bajic VB, Choudhary V, Hock CK (2004). Content analysis of the core promoter region of human genes. In Silico Biol.

[B38] Gomes FC, Sousa Vde O, Romao L (2005). Emerging roles for TGF-beta1 in nervous system development. Int J Dev Neurosci.

[B39] Shi Y, Massague J (2003). Mechanisms of TGF-beta signaling from cell membrane to the nucleus. Cell.

[B40] Miller MW, Luo J (2002). Effects of ethanol and transforming growth factor beta (TGF beta) on neuronal proliferation and nCAM expression. Alcohol Clin Exp Res.

[B41] Chai Y, Ito Y, Han J (2003). TGF-beta signaling and its functional significance in regulating the fate of cranial neural crest cells. Crit Rev Oral Biol Med.

[B42] Ikonomidou C, Bittigau P, Ishimaru MJ, Wozniak DF, Koch C, Genz K, Price MT, Stefovska V, Horster F, Tenkova T, Dikranian K, Olney JW (2000). Ethanol-induced apoptotic neurodegeneration and fetal alcohol syndrome. Science.

[B43] Farber NB, Olney JW (2003). Drugs of abuse that cause developing neurons to commit suicide. Brain Res Dev Brain Res.

[B44] Thiery JP (2003). Cell adhesion in development: a complex signaling network. Curr Opin Genet Dev.

[B45] Wang D, Kanuma T, Mizunuma H, Takama F, Ibuki Y, Wake N, Mogi A, Shitara Y, Takenoshita S (2000). Analysis of specific gene mutations in the transforming growth factor-beta signal transduction pathway in human ovarian cancer. Cancer Res.

[B46] Hahn SA, Schutte M, Hoque AT, Moskaluk CA, da Costa LT, Rozenblum E, Weinstein CL, Fischer A, Yeo CJ, Hruban RH, Kern SE (1996). DPC4, a candidate tumor suppressor gene at human chromosome 18q21.1. Science.

[B47] Garrigue-Antar L, Munoz-Antonia T, Antonia SJ, Gesmonde J, Vellucci VF, Reiss M (1995). Missense mutations of the transforming growth factor beta type II receptor in human head and neck squamous carcinoma cells. Cancer Res.

[B48] Jakowlew SB (2006). Transforming growth factor-beta in cancer and metastasis. Cancer Metastasis Rev.

[B49] Markowitz S, Wang J, Myeroff L, Parsons R, Sun L, Lutterbaugh J, Fan RS, Zborowska E, Kinzler KW, Vogelstein B (1995). Inactivation of the type II TGF-beta receptor in colon cancer cells with microsatellite instability. Science.

[B50] McAllister KA, Grogg KM, Johnson DW, Gallione CJ, Baldwin MA, Jackson CE, Helmbold EA, Markel DS, McKinnon WC, Murrell J (1994). Endoglin, a TGF-beta binding protein of endothelial cells, is the gene for hereditary haemorrhagic telangiectasia type 1. Nat Genet.

[B51] Mashima Y, Yamamoto S, Inoue Y, Yamada M, Konishi M, Watanabe H, Maeda N, Shimomura Y, Kinoshita S (2000). Association of autosomal dominantly inherited corneal dystrophies with BIGH3 gene mutations in Japan. Am J Ophthalmol.

[B52] Saito T, Kinoshita A, Yoshiura Ki, Makita Y, Wakui K, Honke K, Niikawa N, Taniguchi N (2001). Domain-specific mutations of a transforming growth factor (TGF)-beta 1 latency-associated peptide cause Camurati-Engelmann disease because of the formation of a constitutively active form of TGF-beta 1. J Biol Chem.

[B53] Isaka Y, Brees DK, Ikegaya K, Kaneda Y, Imai E, Noble NA, Border WA (1996). Gene therapy by skeletal muscle expression of decorin prevents fibrotic disease in rat kidney. Nat Med.

[B54] Shah M, Foreman DM, Ferguson MW (1995). Neutralisation of TGF-beta 1 and TGF-beta 2 or exogenous addition of TGF-beta 3 to cutaneous rat wounds reduces scarring. J Cell Sci.

[B55] Lee TY, Chin GS, Kim WJ, Chau D, Gittes GK, Longaker MT (1999). Expression of transforming growth factor beta 1, 2, and 3 proteins in keloids. Ann Plast Surg.

[B56] Khalil N, Greenberg AH (1991). The role of TGF-beta in pulmonary fibrosis. Ciba Found Symp.

[B57] Castilla A, Prieto J, Fausto N (1991). Transforming growth factors beta 1 and alpha in chronic liver disease. Effects of interferon alfa therapy. N Engl J Med.

[B58] Das P, Golde T (2006). Dysfunction of TGF-beta signaling in Alzheimer's disease. J Clin Invest.

[B59] Tesseur I, Zou K, Esposito L, Bard F, Berber E, Can JV, Lin AH, Crews L, Tremblay P, Mathews P, Mucke L, Masliah E, Wyss-Coray T (2006). Deficiency in neuronal TGF-beta signaling promotes neurodegeneration and Alzheimer's pathology. J Clin Invest.

[B60] Krens SF, Spaink HP, Snaar-Jagalska BE (2006). Functions of the MAPK family in vertebrate-development. FEBS Lett.

[B61] Orton RJ, Sturm OE, Vyshemirsky V, Calder M, Gilbert DR, Kolch W (2005). Computational modelling of the receptor-tyrosine-kinase-activated MAPK pathway. Biochem J.

[B62] Aroor AR, Shukla SD (2004). MAP kinase signaling in diverse effects of ethanol. Life Sci.

[B63] Kumada T, Lakshmana MK, Komuro H (2006). Reversal of neuronal migration in a mouse model of fetal alcohol syndrome by controlling second-messenger signalings. J Neurosci.

[B64] Ingham PW, McMahon AP (2001). Hedgehog signaling in animal development: paradigms and principles. Genes Dev.

[B65] Chen SY, Periasamy A, Yang B, Herman B, Jacobson K, Sulik KK (2000). Differential sensitivity of mouse neural crest cells to ethanol-induced toxicity. Alcohol.

[B66] Ahlgren SC, Bronner-Fraser M (1999). Inhibition of sonic hedgehog signaling in vivo results in craniofacial neural crest cell death. Curr Biol.

[B67] Cartwright MM, Smith SM (1995). Increased cell death and reduced neural crest cell numbers in ethanol-exposed embryos: partial basis for the fetal alcohol syndrome phenotype. Alcohol Clin Exp Res.

[B68] Ahlgren SC, Thakur V, Bronner-Fraser M (2002). Sonic hedgehog rescues cranial neural crest from cell death induced by ethanol exposure. Proc Natl Acad Sci USA.

[B69] Damberg M (2005). Transcription factor AP-2 and monoaminergic functions in the central nervous system. J Neural Transm.

[B70] Calella AM, Nerlov C, Lopez RG, Sciarretta C, von Bohlen Und Halbach O, Bereshchenko O, Minichiello L (2007). Neurotrophin/Trk receptor signalling mediates C/EBPalpha, -beta and NeuroD recruitment to immediate-early (IE) gene promoters in neuronal cells and requires C/EBPs to induce IE gene transcription. Neural Develop.

[B71] Greene LA, Liu DX, Troy CM, Biswas SC (2007). Cell cycle molecules define a pathway required for neuron death in development and disease. Biochim Biophys Acta.

[B72] Itoh F, Ishizaka Y, Tahira T, Yamamoto M, Miya A, Imai K, Yachi A, Takai S, Sugimura T, Nagao M (1992). Identification and analysis of the ret proto-oncogene promoter region in neuroblastoma cell lines and medullary thyroid carcinomas from MEN2A patients. Oncogene.

[B73] van Genderen C, Okamura RM, Farinas I, Quo RG, Parslow TG, Bruhn L, Grosschedl R (1994). Development of several organs that require inductive epithelial-mesenchymal interactions is impaired in LEF-1-deficient mice. Genes Dev.

[B74] Bataller L, Wade DF, Graus F, Rosenfeld MR, Dalmau J (2003). The MAZ protein is an autoantigen of Hodgkin's disease and paraneoplastic cerebellar dysfunction. Ann Neurol.

[B75] Okamoto S, Sherman K, Bai G, Lipton SA (2002). Effect of the ubiquitous transcription factors, SP1 and MAZ, on NMDA receptor subunit type 1 (NR1) expression during neuronal differentiation. Brain Res Mol Brain Res.

[B76] Kobayashi A, Yamagiwa H, Hoshino H, Muto A, Sato K, Morita M, Hayashi N, Yamamoto M, Igarashi K (2000). A combinatorial code for gene expression generated by transcription factor Bach2 and MAZR (MAZ-related factor) through the BTB/POZ domain. Mol Cell Biol.

[B77] Perrotti D, Melotti P, Skorski T, Casella I, Peschle C, Calabretta B (1995). Overexpression of the zinc finger protein MZF1 inhibits hematopoietic development from embryonic stem cells: correlation with negative regulation of CD34 and c-myb promoter activity. Mol Cell Biol.

[B78] Lange-Dohna C, Zeitschel U, Gaunitz F, Perez-Polo JR, Bigl V, Rossner S (2003). Cloning and expression of the rat BACE1 promoter. J Neurosci Res.

[B79] Tayaramma T, Ma B, Rohde M, Mayer H (2006). Chromatin-remodeling factors allow differentiation of bone marrow cells into insulin-producing cells. Stem Cells.

[B80] Benfante R, Antonini RA, Vaccari M, Flora A, Chen F, Clementi F, Fornasari D (2005). The expression of the human neuronal alpha3 Na+,K+-ATPase subunit gene is regulated by the activity of the Sp1 and NF-Y transcription factors. Biochem J.

[B81] Hsu SH, Hsieh-Li HM, Huang HY, Huang PH, Li H (2005). bHLH-zip transcription factor Spz1 mediates mitogen-activated protein kinase cell proliferation, transformation, and tumorigenesis. Cancer Res.

[B82] Riazi AM, Lee H, Hsu C, Van Arsdell G (2005). CSX/Nkx2.5 modulates differentiation of skeletal myoblasts and promotes differentiation into neuronal cells in vitro. J Biol Chem.

[B83] Tassabehji M, Hammond P, Karmiloff-Smith A, Thompson P, Thorgeirsson SS, Durkin ME, Popescu NC, Hutton T, Metcalfe K, Rucka A, Stewart H, Read AP, Maconochie M, Donnai D (2005). GTF2IRD1 in craniofacial development of humans and mice. Science.

[B84] Handoko HY, Nancarrow DJ, Mowry BJ, McGrath JJ (2006). Polymorphisms in the vitamin D receptor and their associations with risk of schizophrenia and selected anthropometric measures. Am J Hum Biol.

[B85] Dimitroulakos J, Pienkowska M, Sun P, Farooq S, Zielenska M, Squire JA, Yeger H (1999). Identification of a novel zinc finger gene, zf5-3, as a potential mediator of neuroblastoma differentiation. Int J Cancer.

[B86] US National Library of Medicine – PubMed tutorial. http://www.nlm.nih.gov/bsd/disted/pubmed.html.

[B87] Dragon Disease Explorer. http://research.i2r.a-star.edu.sg/DRAGON/DE/.

[B88] Kelso J, Visagie J, Theiler G, Christoffels A, Bardien S, Smedley D, Otgaar D, Greyling G, Jongeneel CV, McCarthy MI, Hide T, Hide W (2003). eVOC: a controlled vocabulary for unifying gene expression data. Genome Res.

[B89] Pan H, Zuo L, Choudhary V, Zhang Z, Leow SH, Chong FT, Huang Y, Ong VW, Mohanty B, Tan SL, Krishnan SP, Bajic VB (2004). Dragon TF Association Miner: a system for exploring transcription factor associations through text-mining. Nucleic Acids Res.

[B90] Cudd TA (2005). Animal model systems for the study of alcohol teratology. Exp Biol Med (Maywood).

[B91] Sulik KK (2005). Genesis of alcohol-induced craniofacial dysmorphism. Exp Biol Med (Maywood).

[B92] Eppig JT, Bult CJ, Kadin JA, Richardson JE, Blake JA, the members of the Mouse Genome Database Group (2005). The Mouse Genome Database (MGD): from genes to mice – a community resource for mouse biology. Nucleic Acids Res.

[B93] Blake JA, Eppig JT, Bult CJ, Kadin JA, Richardson JE (2006). The Mouse Genome Database (MGD): updates and enhancements. Nucleic Acids Res.

[B94] Oti M, Brunner H (2007). The modular nature of genetic diseases. Clin Genet.

[B95] Welch-Carre E (2005). The neurodevelopmental consequences of prenatal alcohol exposure. Adv Neonatal Care.

[B96] Safran M, Chalifa-Caspi V, Shmueli O, Olender T, Lapidot M, Rosen N, Shmoish M, Peter Y, Glusman G, Feldmesser E, Adato A, Peter I, Khen M, Atarot T, Groner Y, Lancet D (2003). Human Gene-Centric Databases at the Weizmann Institute of Science: GeneCards, UDB, CroW 21 and HORDE. Nucleic Acids Res.

[B97] Surani MA (1998). Imprinting and the initiation of gene silencing in the germ line. Cell.

[B98] Delaval K, Feil R (2004). Epigenetic regulation of mammalian genomic imprinting. Curr Opin Genet Dev.

[B99] Lim HN, van Oudenaarden A (2007). A multistep epigenetic switch enables the stable inheritance of DNA methylation states. Nat Genet.

[B100] Valles S, Pitarch J, Renau-Piqueras J, Guerri C (1997). Ethanol exposure affects glial fibrillary acidic protein gene expression and transcription during rat brain development. J Neurochem.

[B101] Garro AJ, McBeth DL, Lima V, Lieber CS (1991). Ethanol consumption inhibits fetal DNA methylation in mice: implications for the fetal alcohol syndrome. Alcohol Clin Exp Res.

[B102] Wagschal A, Feil R (2006). Genomic imprinting in the placenta. Cytogenet Genome Res.

[B103] Glaser RL, Ramsay JP, Morison IM (2006). The imprinted gene and parent-of-origin effect database now includes parental origin of de novo mutations. Nucleic Acids Res.

[B104] Jirtle RL, Sander M, Barrett JC (2000). Genomic imprinting and environmental disease susceptibility. Environ Health Perspect.

[B105] Bajic VB, Tan SL, Christoffels A, Schonbach C, Lipovich L, Yang L, Hofmann O, Kruger A, Hide W, Kai C, Kawai J, Hume DA, Carninci P, Hayashizaki Y (2006). Mice and men: their promoter properties. PLoS Genet.

