# Supplementary material for: Computational selection and prioritization of candidate genes for Fetal Alcohol Syndrome
Source: BMC Genomics. 2007 Oct 25;8:389. doi: 10.1186/1471-2164-8-389 (PMC2194724; doi:10.1186/1471-2164-8-389)
Supplement: Additional file 1 — The 87 top-ranked genes for FAS identified using binary matrix filtering [file 1471-2164-8-389-S1.pdf]

Rank	Criteria Matched	HGNC ID	Description	Locus	Function
1	17/29	FGFR1	Fibroblast growth factor receptor 1	8p11.2	Involved in limb induction, play a role in bone elongation modulation
2	16/29	MSX1	Msh homeobox homolog 1 gene	4p16.3-p16.1	Potential repressor function in cell cycle progression, transcription repressor
3	15/29	FGFR2	Fibroblast growth factor receptor 2	10q26	Involved in vertebral development, important regulator of bone formation and osteoblast activity
4	15/29	FOXG1B	Forkhead box G1B	14q13	Embryonic transcriptional regulator, playing a critical role in brain development
5	15/29	HOXA1	Homeobox A1	7p15.3	Involved in the placement of hindbrain segments in the proper location along the anterior-posterior axis during development
6	14/29	BMP4	Bone morphogenetic protein 4	14q22-q23	Regulating myogenesis through dosage-dependent PAX3 expression in pre-myogenic cells, inducing apoptosis and chondrogenesis in the chick limb bud
7	14/29	FGFR	Fibroblast growth factor receptor 3	4p16.3	Negative regulator of bone growth promotion, inhibition of chondrocyte proliferation and differentiation depending on devlopmental time
8	14/29	GNAS	GNAS complex locus	20q13.2-q13.3	Involved as modulators or transducers in various transmembrane signaling systems primarily mediating the differential effects of parathyroid hormone
9	14/29	PAX6	Paired box gene 6	11p13	Key regulator of eye, pancreas, central nervous system development and regulator of glial precursors in the ventral neural tube
10	13/29	CASP3	Caspase 3	4q35	Effector caspase in the Fas proapoptotic pathway, playing a crucial role during apoptosis
11	13/29	CITED2	Cbp/p300-interacting transactivator 2	6q23.3	Cytokine-inducible transcription factor with transformation activity controlling left-right patterning and heart development
12	13/29	DLX5	Distal-less homeo box 5	7q21.3	Playing a role in forebrain and craniofacial development and in production of gaba-ergic neurons
13	13/29	EGFR	Epidermal growth factor receptor	7p12	Involved in the control of cell growth and differentiation in early craniofacial development and palate closure and in keratinocyte differentiation
14	13/29	GLI2	Zinc finger protein GLI2	2q24	Involved in the formation of lung, trachea and oesophagus, playing a role in head development
15	13/29	GLI3	Zinc finger protein GLI3	7p14.1-p13	Involved in the development of the CNS, craniofacial structure, lung, trachea and oesophagus
16	13/29	IGF2	Insulin-like growth factor 2	11p15.5	Potent mitogen influenced by placental lactogen and may be playing a role in fetal development
17	13/29	ITGA6	Integrin alpha 6 precursor	2q22-q31	Cell surface adhesion receptor mediating cell-adhesion to extra cellular matrix or to other cells, also involved in the fertilization and embryonic development
18	13/29	MECP2	Methyl cpg binding protein 2	Xq28	Involved in the regulation of gene expression (in normal neuronal maturation) and may regulate the transcription genes in neuronal cells, important in synapse development and neuronal plasticity
19	13/29	MEST	Mesoderm specific transcript isoform A	7q32	Mesoderm-specific transcript, expressed in fetal tissues from the paternal chromosome

20	13/29	PAFAH1B1	Platelet-activating factor acetylhydrolase IB subunit alpha	17p13.3	Component of overlapping but distinct signal pathways including DCX, that promotes neuronal migration
21	13/29	PAX3	Paired box gene 3	2q36.1-q36.2	Involved in neurogenesis, in skeletal muscle development and in melanogenesis through MITF transactivation and maybe other processes
22	13/29	PTCH1	Protein patched homolog 1	9q22.32-q22.33	Repressing SMO activity in the absence of SHH, and involved in endocytosis and vesicle transport
23	13/29	PTPN11	Protein tyrosine phosphatase, non-receptor type 11	12q24.1	Involved in intracellular signal transduction in response to PDGF, EGF and insulin
24	13/29	RB1	Retinoblastoma 1	13q14.2	Regulator of cell growth, may function in cell cycle exit, differentiation and survival of hair cells
25	13/29	SOX2	Transcription factor SOX-2	3q26.3-q27	Activating FGF4 and modulator of LINE retroposons promoter activity
26	13/29	TGFB1	Transforming growth factor, beta 1	19q13.2	Stimulating articular chondrocyte cell growth through MAPK3 activation and inducing apoptosis in endothelial cells
27	12/29	CREBBP	CREB binding protein	16p13.3	Playing a pivotal role in embryonic development, involved in a variety of transcriptional pathways through chromatin remodeling
28	12/29	CTNNB1	Catenin beta-1	3p22-p21.3	Multifunctional protein participating in cell-cell adhesion and Wnt-stimulated transcriptional activation and the establishment of a bipolar mitotic spindle
29	12/29	DTNA	Dystrobrevin alpha	18q12.1-q12.2	May be involved in signal transduction in myeloid cells during induction of granulocytic differentiation and/or at the commitment stage of differentiation or phagocytic cells
30	12/29	GABRB3	Gamma-aminobutyric-acid receptor subunit beta-3 precursor	15q11.2-q12	Mediating neuronal inhibition by binding to the gaba-benzodiazepine receptor and opening an integral chloride channel
31	12/29	JAG1	Jagged 1 precursor	20p12.1-p11.23	NOTCH1 ligand playing a pivotal role in the development of the organ of Corti and specification of some vestibular sensory epithelia
32	12/29	MET	Hepatocyte growth factor receptor precursor	7q31.2-q31.3	Important regulator of cell proliferation and differentiation, organ regeneration, embryogenesis and tumorogenesis
33	12/29	NGFR	Nerve growth factor receptor	17q21.31	Playing a central role for mediating inhibitory signals from CNS myelin
34	12/29	PITX2	Pituitary homeobox 2	4q25-q27	RNA polymerase 2 transcription factor, involved in the regulation of heart
35	12/29	PXMP3	Peroxisomal membrane protein 3	8q21.1	Implicated in the biogenesis of peroxisomes
36	12/29	SIX3	Homeobox protein SIX3	2p21-p16	Involved in regulation of eye and neural plate development
37	12/29	SMAD2	Mothers against decapentaplegic homolog	18q21	Intracellular mediator of TGFB family of cytokines and activin type 1 receptor
38	12/29	SNAP25	Synaptosomal associated protein 25	20p12-p11.2	May play an important role in the synaptic function of specific neuronal systems
39	12/29	UBE3A	Ubiquitin protein ligase E3A	15q12	Playing a major role in neural development, role in regulating growth of neuronal processes or synapse formation
40	12/29	VEGF-A	Vascular endothelial growth factor	6p12	Key regulator of blood vessel growth, and protecting endothelial cells from apoptosis

41	11/29	AKT1	RAC-alpha serine/threonine-protein kinase	14q32.32	A key regulator for cell growth, cell survival and metabolic insulin action
42	11/29	APP	Amyloid beta A4 protein precursor	21q21.2	Playing a significant role in regulating cerebral thrombosis and increases can profoundly enhance cerebral hemorrhage
43	11/29	ARNT	Aryl hydrocarbon receptor nuclear translocator	1q21	Dioxin receptor translocator, activating genes involved in metabolism, angiogenesis and apoptosis
44	11/29	BCL2L1	BCL2-like 1 Apoptosis regulator Bcl-X	20q11.1	Involved in regulation of cell death by blocking the voltage dependent anion channel, # has a role in preventing BAX activation at the mitochondrial membrane
45	11/29	BMP7	Bone morphogenetic protein 7	20q13.2	Stimulating initial dendritic growth in sympathetic neurons, important regulator of cell development and differentiation of various organs
46	11/29	BRCA1	Breast cancer 1	17q21	Negative regulator of mammary cell growth, having critical function in the proliferation and differentiation of neural progenitor cells
47	11/29	CDKN1B	Cyclin-dependent kinase inhibitor 1B	12p13.1-p12	Critical terminal effectors of signal transduction pathways that control cell differentiation, mediating cell cycle regulation by AFX-like forkhead transcription factors
48	11/29	COL2A1	Collagen, type II, alpha 1	12q13.1	Involved in the structure of cartilage collagen
49	11/29	CXCR4	Chemokine receptor type 4	2q21	Allows cells to migrate in response to a gradient of chemokine ligands, playing a role of receptor for SDF1 to directing the primordial germ cell migration
50	11/29	DCN	Decorin precursor	12q13.2	Playing a role in epithelial/mesenchymal interactions during organ development and shaping, playing a role in matrix assembly
51	11/29	EDG2	Lysophosphatidic acid receptor Edg-2	9q31.3-q32	Involved in the control of myelination, activating phospholipase C
52	11/29	EMX2	Homeobox protein EMX2	10q26.1	Involved in brain development, normal growth and maturation of hippocampus
53	11/29	FGF2	Fibroblast growth factor 2	4q26-q27	Inducer of anteroposterior neural pattern, essential for FGF8 and FGF10 reciprocal regulation in limb induction in mouse and involved in angiogenesis
54	11/29	GDF11	Growth differentiation factor 11	12q12	Involved in mesodermal formation and neurogenesis during embryonic development
55	11/29	GJA1	Gap junction protein, alpha 1	6q22.3	Member of the connexin of intercellular channels, providing a route for the diffusion of materials of low molecular weight from cell to cell
56	11/29	GTF2I	General transcription factor II-I	7q11.23	Multifunctional phosphoprotein with roles in transcription and signal transduction
57	11/29	HMGB1	High mobility group protein B1	13q12	DNA-binding protein, regulating gene transcription and stabilizing nucleosome formation
58	11/29	HOXB2	Homeobox protein Hox-B2	17q21.3	developmental regulatory system transcription factor that provide cells positional identities on the anterior-posterior axis
59	11/29	LDB1	LIM domain-binding protein 1	10q24-q25	Required for LIM-homeodomain proteins to exert their biological activities
60	11/29	LHX2	LIM-homeobox domain protein	9q33-q34.1	Acting as a tissue specific transcriptional activator of the alpha subunit of glycoprotein hormones, involved in the control of cell differentiation in developing lymphoid and neural cell types

61	11/29	LIMK1	LIM motif-containing protein kinase	7q11.23	May act as a link between stress-induced ceramide formation and reorganization of the actin cytoskeleton
62	11/29	LMO4	LIM domain only 4	1p22.3	Transcriptional regulator participating to mammary gland development, repressor of BRCA1
63	11/29	MAPK3	Mitogen-activated protein kinase 3	16p11.2	Activation of MAPK3 is a key regulator of the increased transition to hypertrophic differentiation of the growth plate
64	11/29	MARCKS	Myristoylated alanine-rich protein kinase C substrate	6q21-q22.2	Actin filament cross linking protein regulator of actin cytoskeleton
65	11/29	NCAM1	Neural cell adhesion molecule 1	11q23.1	Involved in neuron-neuron adhesion, neurite fasciculation, outgrowth of neurites
66	11/29	NF1	Neurofibromin 1	17q11.2	Required in endothelial cells but do not rule out a simultaneous requirement in the neural crest during cardiac development
67	11/29	NPAS3	Neuronal PAS domain protein 3	14q12-q13	Neuronal transcription factor
68	11/29	NR2F1	COUP transcription factor 1	5q14	Nuclear receptor involved in the organogenesis, transcription factor
69	11/29	NR2F2	COUP transcription factor 2	15q26.1-q26.2	May be required for angiogenesis and heart development, negative post transcriptional regulator of MYOD1 function
70	11/29	NR2F6	Orphan nuclear receptor EAR-2	19p13.1	Nuclear receptor involved in action regulation of the transcription of GNRH1 gene
71	11/29	OTX1	Homeobox protein OTX1	2p13	Required for sense organ development. Required for the refinement of exuberant axonal projections to subcortical targets
72	11/29	PBX1	Pre-B-cell leukemia transcription factor 1	1q23	May be playing a role in steroidogenesis, sexual development, and megakaryocytic gene expression
73	11/29	BMI1	Polycomb group RING finger protein 4	10p13	Involved in maintaining the transcriptional repressive state of genes, playing an essential role for the generation of self-renewing adult hematopoietic stem cells
74	11/29	PDGFRA	Platelet-derived growth factor receptor, alpha	4q11-q12	Growth arrest specific gene (gas), subunit, receptor tyrosine kinase, class III, binding both PDGFA and PDGFB and having a tyrosine-protein kinase activity
75	11/29	PHC1	Polyhomeotic-like protein 1	12p13	Can play a key role in organogenesis by helping to maintain the expression of a selector gene
76	11/29	PHC2	Polyhomeotic-like protein 2	1p34.3	Required to maintain transcriptional repressive state of many genes, incl. HOX genes, during development
77	11/29	PPARBP	Peroxisome proliferator-activated receptor-binding protein	17q12-q21.1	Essential role for embryonic fibroblast differentiation pathway, and normal development of vital organ systems
78	11/29	RARG	Retinoic acid receptor, gamma	12q13.13	Ligand activated transcription factor
79	11/29	RXRA	Retinoid X receptor, alpha	9q34.3	Involved in retinoic acid response pathway, regulates cardiac morphogenesis
80	11/29	SOX11	Transcription factor SOX-11	2p25.3	Playing a role in the developing nervous system
81	11/29	SPRY2	Sprouty homolog 2	13q31-q32	Required for growth factor stimulated translocation of the protein to membrane ruffles
82	11/29	TGFB2	Transforming growth factor, beta 2	1q41	Having suppressing effects on interleukin-2 dependent T-cell growth

83	11/29	TP53	Tumor suppressor p53	17p13.1	Transcriptional activator through acetylation of transactivation site by CREBBP, activator of target genes promoting growth arrest or cell death in response to DNA damage
84	11/29	TWIST1	Twist-related protein 1	7p21.2	Regulator of embryonic morphogenesis and playing an essential role in metastasis by promoting an epithelial-mesenchymal transition
85	11/29	WNT5A	Protein Wnt-5a precursor	3p21.1	Modulating cell fate and cell behavior during vertebrate development
86	11/29	YWHAE	14-3-3 protein epsilon	17p13.3	Inhibitor of apoptosis through inhibiting the activation of p38 MAP kinase, multifunctional regulator required for cytoplasmic dynein function, neuronal migration and brain development
87	11/29	ZIC2	Zic family member 2	13q32	Putative regulator of the kinetic neural development
