# Supplementary material for: Computational selection and prioritization of candidate genes for Fetal Alcohol Syndrome
Source: BMC Genomics. 2007 Oct 25;8:389. doi: 10.1186/1471-2164-8-389 (PMC2194724; doi:10.1186/1471-2164-8-389)
Supplement: Additional file 2 — GO term annotations significantly over-represented among the top-ranked genes. The table provided represent the GO biological process, cellular component and molecular function terms over-represented among the top-ranked genes [file 1471-2164-8-389-S2.pdf]

GO Biological Process Term	Count	*P*-value[1]
development	53	$4.5x10^{-23}$
organ development	28	$2.9x10^{-17}$
regulation of cellular process	55	$3.9x10^{-13}$
regulation of biological process	56	$1.7x10^{-12}$
regulation of physiological process	53	$2.5x10^{-12}$
morphogenesis	25	$2.9x10^{-12}$
regulation of cellular physiological process	52	$3.8x10^{-12}$
regulation of transcription DNA-dependent	39	$2.3x10^{-11}$
transcription DNA-dependent	39	$7.4x10^{-11}$
regulation of cellular metabolism	41	$9.6x10^{-11}$
regulation of transcription	39	$1.5x10^{-10}$
regulation of nucleic acid metabolism 39 44.8%	39	$2.5x10^{-10}$
regulation of metabolism	41	$3.2x10^{-10}$
skeletal development	12	$3.9x10^{-10}$
transcription	39	$6.9x10^{-10}$
system development	20	$1.0x10^{-09}$
organ morphogenesis	14	$2.8x10^{-09}$
nervous system development	19	$6.3x10^{-09}$
cell communication	44	$5.1x10^{-8}$
signal transduction	41	$1.3x10^{-7}$
transcription from RNA polymerase II promoter	17	$8.3x10^{-7}$
cell differentiation	16	0.0000017
regulation of transcription from RNA polymerase II promoter	12	0.0000076
Growth	10	0.000011
positive regulation of cellular process	16	0.000016
positive regulation of cellular metabolism	9	0.000024
embryonic development	7	0.000027
nucleic acid metabolism	40	0.000032
positive regulation of cellular physiological process	14	0.000039
positive regulation of metabolism	9	0.000052
pattern specification	5	0.00006
positive regulation of physiological process	14	0.000063
mesoderm development	5	0.000072
enzyme linked receptor protein signaling pathway	9	0.000089

positive regulation of biological process	16	0.000089
positive regulation of transcription, DNA-dependant	7	0.00013
brain development	5	0.00014
negative regulation of cellular process	16	0.00038
negative regulation of cellular physiological process	15	0.00045
positive regulation of transcription	7	0.00049
negative regulation of physiological process	15	0.00055
cell proliferation	13	0.00056
transmembrane receptor protein tyrosine kinase signaling pathway	7	0.00058
positive regulation of nucleic acid metabolism	7	0.00058
tissue development	7	0.00065
negative regulation of biological process	16	0.00081
regulation of progression through cell cycle	12	0.0011
segmentation	3	0.0011
regulation of cell cycle	12	0.0011
regulation of cell size	7	0.0011
cell growth	7	0.0011
apoptosis	13	0.0012
programmed cell death	13	0.0012
central nervous system development	6	0.0013
cell death	13	0.0016
death	13	0.0018
angiogenesis	5	0.002
blood vessel morphogenesis	5	0.0024
blood vessel development	5	0.0024
vasculature development	5	0.0024
embryonic pattern specification	3	0.0029
neurophysiological process	11	0.0031
cell surface receptor linked signal transduction	16	0.0034
androgen receptor signaling pathway	4	0.0045
regulation of apoptosis	9	0.0063
regulation of programmed cell death	9	0.0064
regulation of cell proliferation	8	0.0071
steroid hormone receptor signaling pathway	4	0.0073

cell cycle	13	0.0083
intracellular receptor-mediated signaling pathway	4	0.0084
negative regulation of progression through cell cycle	6	0.0097
protein amino acid phosphorylation	11	0.01
negative regulation of transcription, DNA-dependent	5	0.011
cell motility	7	0.011
locomotion	7	0.011
localization of cell	7	0.011
positive regulation of cell proliferation	5	0.015
cellular morphogenesis	7	0.02
ossification	3	0.02
biomineral formation	3	0.02
periodic pa itioning	2	0.021
segment polarity determination	2	0.021
negative regulation of transcription from RNA polymerase II promoter	4	0.022
bone remodeling	3	0.022
tissue remodeling	3	0.022
cell organization and biogenesis	19	0.023
primary metabolism	57	0.025
striated muscle development	3	0.026
negative regulation of apoptosis	5	0.028
negative regulation of programmed cell death	5	0.029
muscle development	4	0.031
positive regulation of transcription from RNA polymerase II promoter	3	0.037
phosphorylation	11	0.038
negative regulation of transcription	5	0.038
blastoderm segmentation	2	0.041
sensory perception of sound	4	0.043
sensory perception of mechanical stimulus	4	0.043
negative regulation of nucleic acid metabolism	5	0.046
cell cycle checkpoint	3	0.047
cell migration	4	0.049
cell-cell signaling	8	0.049
positive regulation of organismal physiological process	3	0.049

GO Cellular Component Term	Count	P-value[1]
Nucleus	46	$5.6x10^{-6}$
extracellular region	15	0.004
extracellular space	7	0.042

GO Molecular Function Term	Count	P-value[1]
transcription regulator activity	36	$4.6x10^{-15}$
transcription factor activity	29	$1.4x10^{-13}$
DNA binding	39	$1.6x10^{-11}$
sequence-specific DNA binding	18	$9.3x10^{-11}$
protein binding	59	$1.7x10^{-09}$
signal transducer activity	37	$8.3x10^{-9}$
transcription factor binding	15	$1.1x10^{-7}$
nucleic acid binding	39	$1.5x10^{-6}$
growth factor activity	9	$2.4x10^{-6}$
transcription cofactor activity	12	$4.9x10^{-6}$
transcriptional activator activity	11	$8.2x10^{-06}$
Binding	78	0.00001
transcription coactivator activity	9	0.000035
receptor binding	14	0.000059
heparin binding	6	0.00013
steroid hormone receptoractivity	5	0.00041
ligand-dependent nuclear receptor activity	5	0.00051
glycosaminoglycan binding	6	0.00054
fibroblast growth factor receptor activity	3	0.00057
polysaccharide binding	6	0.00065
pattern binding	6	0.00089
receptor activity	18	0.0019
tumor suppressor	3	0.0026
protein-tyrosine kinase activity	7	0.0029
obsolete molecular function	8	0.0046
cell cycle regulator	3	0.006
nuclear hormone receptor binding	4	0.0092

hormone receptor binding	4	0.0092
transmembrane receptor activity	11	0.011
double-stranded DNA binding	3	0.013
androgen receptor binding	3	0.016
carbohydrate binding	6	0.017
RNA polymerase II transcription factor activity	6	0.018
cytokine activity	5	0.018
steroid hormone receptor binding	3	0.025
zinc ion binding	20	0.028
identical protein binding	5	0.041
transforming growth factor beta receptor binding	2	0.048
