# Supplementary material for: Computational selection and prioritization of candidate genes for Fetal Alcohol Syndrome
Source: BMC Genomics. 2007 Oct 25;8:389. doi: 10.1186/1471-2164-8-389 (PMC2194724; doi:10.1186/1471-2164-8-389)
Supplement: Additional file 3 — Promoter element binding site analysis. The tables provided represent the promoter elements that have been found in the target promoter set relative to the background promoter set (Table 1) and the pairs of promoter elements at maximum mutual distance of 50 nucleotides (Table 2) that have been found in the target promoter set relative to the background promoter set. [file 1471-2164-8-389-S3.pdf]

Table 1: The promoter elements that have been found in the target promoter set relative to the background promoter set.

Promoter Elements	ORI	TAR (%)	BCG (%)	Probability of finding PE in target set	Probability of finding PE in background set	TAR (n)	BCG (n)	TAR Total	BCG Total	*P*-value
+1 SRF	4.9832	6.99	3.41	0.00010	0.00004	38	350	544	10255	0.067
-1 Nkx6-2	3.8034	7.17	3.6	0.00006	0.00003	39	369	544	10255	0.091
-1 IPF1	3.3286	5.51	2.98	0.00005	0.00003	30	306	544	10255	1.000
-1 SRF	3.1818	6.25	3.81	0.00009	0.00005	34	391	544	10255	1.000
+1 TBX5	3.0911	7.9	4.45	0.00007	0.00004	43	456	544	10255	0.406
+1 Pit-1	3.0386	5.33	3.15	0.00005	0.00003	29	323	544	10255	1.000
+1 HSF1	2.7461	8.64	5.03	0.00007	0.00005	47	516	544	10255	0.448
+1 HMG IY	2.7395	11.95	7.1	0.00010	0.00006	65	728	544	10255	0.060
+1 IRF1	2.6653	12.68	7.93	0.00012	0.00007	69	813	544	10255	0.147
+1 MEF-2	2.5417	7.9	5.02	0.00007	0.00004	43	515	544	10255	1.000
-1 Hand1:E47	2.4874	5.7	3.62	0.00005	0.00003	31	371	544	10255	1.000
+1 Cdx-2	2.3874	10.85	7.11	0.00010	0.00006	59	729	544	10255	1.000
+1 C/EBPgamma	2.3669	7.9	5.03	0.00007	0.00004	43	516	544	10255	1.000
-1 Sp3	2.2802	6.25	4.08	0.00005	0.00004	34	418	544	10255	1.000
-1 Pbx-1	2.2091	15.07	10.13	0.00014	0.00009	82	1039	544	10255	0.315
-1 DBP	2.1563	9.93	6.57	0.00008	0.00006	54	674	544	10255	1.000
+1 C/EBPdelta	2.1086	5.15	3.52	0.00004	0.00003	28	361	544	10255	1.000
+1 NF-AT	2.0871	5.7	4.16	0.00006	0.00004	31	427	544	10255	1.000
-1 TBP	2.0639	9.38	6.49	0.00008	0.00006	51	666	544	10255	1.000

Table 2: Pairs of PE at maximum mutual distance of 50 nucleotides that have been found in the target promoter set relative to the background promoter set.

Pairs of promoter elements	ORI	TAR (%)	BCG (%)	Probability of finding PE in target set	Probability of finding PE in background set	TAR (n)	BCG (n)	TAR Total	BCG Total	*P*-value
-1 LBP-1/+1 AP-2	10.7173	5.15	1.64	0.00006	0.00002	28	168	544	10255	0.618
+1 Pax-4/+1 FAC1	9.354	5.33	1.84	0.00006	0.00002	29	189	544	10255	1.000
-1 Spz1/-1 ETF	8.8907	6.43	2.71	0.00014	0.00004	35	278	544	10255	1.000
-1 MZF1/-1 VDR	8.3078	7.9	3.15	0.00017	0.00005	43	323	544	10255	0.194
+1 FAC1/+1 Pax-4	7.8354	5.15	2.3	0.00010	0.00003	28	236	544	10255	1.000
-1 MAZ/+1 ZF5	7.7859	6.25	2.04	0.00006	0.00002	34	209	544	10255	0.062
+1 Pax-4/-1 MZF1	7.6641	5.51	2.4	0.00008	0.00003	30	246	544	10255	1.000
-1 ZF5/-1 MAZ	7.2777	6.62	2.94	0.00011	0.00004	36	301	544	10255	1.000
+1 ETF/-1 Sp1	6.6357	6.8	4.17	0.00037	0.00009	37	428	544	10255	1.000
-1 ZF5/+1 C/EBP	6.2165	5.15	2.11	0.00006	0.00002	28	216	544	10255	1.000
-1 MAZ/+1 AP-2	5.9377	5.7	2.38	0.00008	0.00003	31	244	544	10255	1.000
+1 C/EBP/+1 Spz1	5.8262	6.07	2.44	0.00006	0.00003	33	250	544	10255	1.000
+1 Sp1/-1 Spz1	5.7749	10.85	5.45	0.00030	0.00010	59	559	544	10255	1.000
+1 ZF5/+1 MAZ	5.3521	6.8	2.78	0.00008	0.00004	37	285	544	10255	1.000
+1 ZF5/-1 MAZ	5.171	5.33	2.15	0.00005	0.00003	29	220	544	10255	1.000
-1 VDR/-1 MAZ	5.1338	6.8	3.17	0.00014	0.00006	37	325	544	10255	1.000
+1 ZF5/-1 AP-2gamma	4.9973	5.33	2.33	0.00005	0.00002	29	239	544	10255	1.000
+1 E2F/-1 VDR	4.9316	7.17	4.21	0.00015	0.00005	39	432	544	10255	1.000
+1 Spz1/+1 Spz1	4.903	8.82	5.14	0.00021	0.00007	48	527	544	10255	1.000
-1 Spz1/-1 MAZ	4.8704	5.7	2.72	0.00009	0.00004	31	279	544	10255	1.000
+1 Pax-4/-1 Spz1	4.8571	9.93	4.85	0.00012	0.00005	54	497	544	10255	1.000
-1 VDR/+1 VDR	4.8498	6.62	3.21	0.00009	0.00004	36	329	544	10255	1.000
+1 Oct-1/+1 Pax-4	4.706	6.43	2.96	0.00008	0.00003	35	304	544	10255	1.000
+1 Spz1/-1 Pax-4	4.6875	9.01	4.85	0.00012	0.00005	49	497	544	10255	1.000
-1 ETF/+1 E2F	4.5575	6.8	3.66	0.00011	0.00005	37	375	544	10255	1.000
-1 MAZ/-1 Spz1	4.5301	5.33	2.37	0.00007	0.00004	29	243	544	10255	1.000
-1 Spz1/+1 VDR	4.5179	6.07	2.75	0.00007	0.00003	33	282	544	10255	1.000
-1 MZF1/-1 ZF5	4.4631	5.7	2.79	0.00007	0.00003	31	286	544	10255	1.000
-1 Spz1/+1 Pax-4	4.4298	9.01	4.85	0.00012	0.00005	49	497	544	10255	1.000
+1 Spz1/+1 VDR	4.3932	11.76	6.92	0.00027	0.00010	64	710	544	10255	1.000
+1 ETF/-1 ZF5	4.2011	9.93	6.5	0.00029	0.00011	54	667	544	10255	1.000
-1 Oct-1/+1 Pax-4	4.1981	6.8	3.16	0.00007	0.00004	37	324	544	10255	1.000
+1 ETF/+1 Sp1	4.1285	8.09	5.8	0.00061	0.00021	44	595	544	10255	1.000
-1 VDR/+1 Pax-4	4.1164	10.29	5.88	0.00018	0.00008	56	603	544	10255	1.000
-1 Pax-4/-1 Pax-2	4.0956	5.15	2.55	0.00005	0.00002	28	262	544	10255	1.000
+1 AP-2/-1 ETF	4.0559	9.19	5.3	0.00023	0.00010	50	544	544	10255	1.000
-1 C/EBP/-1 GEN_INI	4.0546	6.99	3.43	0.00014	0.00007	38	352	544	10255	1.000
-1 Pax-4/-1 Oct-1	4.0195	6.07	3.11	0.00007	0.00004	33	319	544	10255	1.000
-1 Tst-1/+1 Pax-4	4.0172	5.33	2.54	0.00005	0.00002	29	260	544	10255	1.000
-1 ETF/+1 ZF5	3.8938	9.38	5.66	0.00018	0.00008	51	580	544	10255	1.000
-1 ETF/-1 ZF5	3.7955	11.03	6.13	0.00022	0.00011	60	629	544	10255	1.000
-1 C/EBP/-1 Pax-4	3.6539	9.38	4.84	0.00009	0.00005	51	496	544	10255	1.000
+1 ETF/+1 AP-2	3.6306	9.19	6.51	0.00030	0.00011	50	668	544	10255	1.000
+1 C/EBP/+1 GEN_INI	3.5705	7.54	3.92	0.00016	0.00009	41	402	544	10255	1.000
-1 C/EBP/-1 Oct-1	3.5228	5.51	3.46	0.00009	0.00004	30	355	544	10255	1.000

+1 ZF5/-1 ETF	3.4419	10.85	5.69	0.00016	0.00009	59	583	544	10255	1.000
-1 Spz1/-1 AP-2	3.4355	8.27	5.38	0.00017	0.00008	45	552	544	10255	1.000
-1 VDR/-1 MZF1	3.4345	5.88	3.22	0.00009	0.00005	32	330	544	10255	1.000
-1 VDR/-1 VDR	3.2937	15.07	8.08	0.00028	0.00016	82	829	544	10255	0.130
+1 ETF/+1 ETF	3.2705	10.85	8.06	0.00062	0.00025	59	827	544	10255	1.000
-1 E2F/-1 ETF	3.2424	7.72	5.54	0.00022	0.00009	42	568	544	10255	1.000
+1 ZF5/-1 VDR	3.2332	9.93	6.35	0.00017	0.00008	54	651	544	10255	1.000
+1 VDR/+1 MAZ	3.2282	6.43	2.83	0.00006	0.00005	35	290	544	10255	1.000
-1 VDR/-1 E2F	3.211	9.38	6.46	0.00021	0.00009	51	662	544	10255	1.000
-1 MAZ/-1 MAZ	3.1829	5.15	1.49	0.00006	0.00006	28	153	544	10255	0.113
-1 MZF1/+1 Sp1	3.0484	5.7	2.37	0.00006	0.00005	31	243	544	10255	1.000
+1 Sp1/-1 VDR	3.0235	11.76	7.01	0.00028	0.00016	64	719	544	10255	1.000
-1 Oct-1/-1 Pax-4	3.0033	6.07	3.43	0.00007	0.00004	33	352	544	10255	1.000
+1 AP-2/+1 GEN_INI	2.9689	6.62	3.68	0.00015	0.00009	36	377	544	10255	1.000
-1 ZF5/+1 ETF	2.9515	9.56	7.01	0.00024	0.00011	52	719	544	10255	1.000
-1 ZF5/-1 ETF	2.916	12.5	7.22	0.00022	0.00013	68	740	544	10255	1.000
-1 Spz1/+1 Sp1	2.8755	10.48	6.07	0.00019	0.00011	57	622	544	10255	1.000
+1 Sp1/-1 MAZ	2.8744	5.7	2.76	0.00009	0.00006	31	283	544	10255	1.000
-1 E2F/+1 ETF	2.8346	5.15	4.59	0.00016	0.00006	28	471	544	10255	1.000
-1 Sp1/-1 MAZ	2.827	5.88	2.94	0.00013	0.00010	32	301	544	10255	1.000
-1 VDR/+1 E2F	2.7991	8.09	4.87	0.00011	0.00007	44	499	544	10255	1.000
-1 Spz1/-1 Sp1	2.7847	12.32	6.63	0.00031	0.00021	67	680	544	10255	1.000
-1 C/EBP/+1 Oct-1	2.7834	6.07	3.59	0.00008	0.00005	33	368	544	10255	1.000
-1 Spz1/-1 E2F	2.7777	8.46	5.56	0.00012	0.00006	46	570	544	10255	1.000
-1 MAZ/-1 Sp1	2.7765	5.51	2.96	0.00014	0.00009	30	304	544	10255	1.000
-1 Pax-4/+1 Oct-1	2.7422	5.33	3.25	0.00006	0.00004	29	333	544	10255	1.000
-1 E2F-1/-1 Pax-4	2.7262	6.8	4.85	0.00011	0.00006	37	497	544	10255	1.000
-1 ZF5/-1 VDR	2.7201	13.42	8.45	0.00022	0.00013	73	867	544	10255	1.000
-1 Spz1/+1 ZF5	2.7194	9.38	5.53	0.00010	0.00006	51	567	544	10255	1.000
-1 VDR/+1 AP-2	2.7174	12.13	7.86	0.00023	0.00013	66	806	544	10255	1.000
-1 Spz1/+1 AP-2	2.6995	8.27	6.1	0.00017	0.00008	45	626	544	10255	1.000
+1 VDR/-1 E2F-1	2.636	5.33	3.97	0.00011	0.00006	29	407	544	10255	1.000
-1 Pax-4/+1 GEN_INI	2.6326	8.82	4.98	0.00016	0.00011	48	511	544	10255	1.000
+1 E2F/+1 ETF	2.6312	11.4	7.67	0.00023	0.00013	62	787	544	10255	1.000
+1 VDR/-1 AP-2	2.5728	10.48	7.78	0.00023	0.00012	57	798	544	10255	1.000
+1 C/EBP/-1 C/EBP	2.5384	8.09	5.33	0.00011	0.00006	44	547	544	10255	1.000
+1 ETF/+1 E2F	2.4797	9.19	7.35	0.00024	0.00012	50	754	544	10255	1.000
-1 Pax-4/+1 Spz1	2.4614	7.35	5.84	0.00012	0.00006	40	599	544	10255	1.000
+1 CDX/+1 CDX	2.433	5.15	2.08	0.00005	0.00005	28	213	544	10255	1.000
-1 Pax-4/+1 VDR	2.4297	8.64	6.38	0.00014	0.00008	47	654	544	10255	1.000
+1 GC box/+1 Spz1	2.397	5.33	3.68	0.00007	0.00004	29	377	544	10255	1.000
+1 C/EBP/+1 C/EBP	2.3729	7.54	4.87	0.00009	0.00006	41	499	544	10255	1.000
-1 ETF/-1 ETF	2.3364	6.62	5.26	0.00036	0.00019	36	539	544	10255	1.000
-1 AP-2/-1 Pax-4	2.3118	8.82	6.24	0.00013	0.00008	48	640	544	10255	1.000
-1 Pax-4/-1 ZF5	2.3093	9.38	6.32	0.00011	0.00007	51	648	544	10255	1.000
+1 ZF5/+1 Spz1	2.3087	10.48	7.54	0.00015	0.00009	57	773	544	10255	1.000
-1 AP-2/+1 AP-2	2.2735	17.1	12.66	0.00043	0.00026	93	1298	544	10255	1.000
-1 E2F-1/+1 Sp1	2.2487	8.09	5.81	0.00030	0.00019	44	596	544	10255	1.000
+1 C/EBP/-1 Pax-4	2.2468	8.09	5.1	0.00008	0.00005	44	523	544	10255	1.000
-1 C/EBP/-1 C/EBP	2.2411	7.54	4.79	0.00008	0.00006	41	491	544	10255	1.000

-1 E2F/+1 AP-2	2.2048	13.6	9.97	0.00028	0.00017	74	1022	544	10255	1.000
+1 E2F/-1 ETF	2.1983	5.51	3.8	0.00007	0.00005	30	390	544	10255	1.000
+1 AP-2/+1 AP-2	2.1656	15.99	12.96	0.00048	0.00027	87	1329	544	10255	1.000
-1 AP-2/-1 AP-2	2.1535	14.52	12.75	0.00055	0.00029	79	1308	544	10255	1.000
+1 ZF5/+1 ETF	2.1293	12.32	9.63	0.00029	0.00018	67	988	544	10255	1.000
+1 AP-2/+1 E2F	2.1068	15.62	11.86	0.00028	0.00018	85	1216	544	10255	1.000
+1 E2F/-1 GC box	2.0944	5.51	3.52	0.00006	0.00005	30	361	544	10255	1.000
-1 Pax-4/-1 C/EBP	2.0906	7.9	5.62	0.00009	0.00006	43	576	544	10255	1.000
+1 ETF/+1 ZF5	2.0791	8.46	9.01	0.00033	0.00015	46	924	544	10255	1.000
+1 AP-2/+1 ZF5	2.0762	20.22	15.76	0.00045	0.00028	110	1616	544	10255	1.000
-1 ZF5/-1 Spz1	2.0761	7.54	6.39	0.00014	0.00008	41	655	544	10255	1.000
+1 Pax-4/-1 C/EBP	2.071	8.27	5.61	0.00009	0.00006	45	575	544	10255	1.000
-1 ZF5/+1 AP-2	2.0443	17.1	14.69	0.00048	0.00027	93	1506	544	10255	1.000
-1 Spz1/+1 GEN_INI	2.0393	5.7	3.77	0.00011	0.00008	31	387	544	10255	1.000
+1 VDR/+1 AP-2	2.0253	9.93	7.66	0.00017	0.00011	54	786	544	10255	1.000
+1 ETF/-1 AP-2	2.0252	8.46	7.29	0.00024	0.00014	46	748	544	10255	1.000
+1 AP-2/-1 AP-2	2.0129	17.1	13.32	0.00040	0.00025	93	1366	544	10255	1.000
+1 ZF5/-1 Pax-4	2.0116	10.48	8.16	0.00015	0.00010	57	837	544	10255	1.000
+1 VDR/+1 Spz1	2.0033	7.9	7.03	0.00019	0.00011	43	721	544	10255	1.000
+1 Pax-4/+1 VDR	2.0013	6.07	4.71	0.00008	0.00005	33	483	544	10255	1.000
